# Anodic titania nanotubes decorated with gold nanoparticles produced by laser-induced dewetting of thin metallic films

**DOI:** 10.1038/s41598-020-77710-x

**Published:** 2020-11-25

**Authors:** Katarzyna Grochowska, Nikolay Nedyalkov, Jakub Karczewski, Łukasz Haryński, Gerard Śliwiński, Katarzyna Siuzdak

**Affiliations:** 1 Centre of Plasma and Laser Engineering, The Szewalski Institute of Fluid-Flow Machinery, Polish Academy of Sciences, 14 Fiszera St., 80-231 Gdańsk, Poland; 2 Institute of Electronics, Bulgarian Academy of Sciences, 72 Tsarigradsko Shose Blvd., 1784 Sofia, Bulgaria; 3 Faculty of Applied Physics and Mathematics, Gdańsk University of Technology, 11/12 Narutowicza St., 80-233 Gdańsk, Poland

**Keywords:** Engineering, Materials for devices, Nanoscale materials, Electrochemistry, Porous materials

## Abstract

Herein, we combine titania layers with gold species in a laser-supported process and report a substantial change of properties of the resulting heterostructures depending on the major processing parameters. Electrodes were fabricated via an anodisation process complemented with calcination to ensure a crystalline phase, and followed by magnetron sputtering of metallic films. The obtained TiO_2_ nanotubes with deposited thin (5, 10 nm) Au films were treated with a UV laser (355 nm) to form Au nanoparticles on top of the nanotubes. It was proven that selected laser working parameters ensure not only the formation of Au nanoparticles, but also simultaneously provide preservation of the initial tubular architecture, while above-threshold laser fluences result in partial destruction (melting) of the top layer of the nanotubes. For almost all of the samples, the crystalline phase of the nanotubes observed in Raman spectra was maintained independently of the laser processing parameters. Enhanced photoresponse up to ca 6 mA/cm^2^ was demonstrated by photoelectrochemical measurements on samples obtained by laser annealing of the 10 nm Au coating on a titania support. Moreover, a Mott–Schottky analysis indicated the dramatically increased (two orders of magnitude) concentration of donor density in the case of a laser-treated Au–TiO_2_ heterojunction compared to reference electrodes.

## Introduction

Novel and efficient devices taking advantage of renewable energy sources, supercapacitors, water-splitting devices and high-capacity batteries, as well as various sensors, are hot topics within the society, and at the same time are challenges for the scientific community. The latest approaches and dynamic development in those fields benefit from materials research at the nanometre-size level. Among the very rich world of functional nanomaterials, one may find quantum dots^1^, metal nanoparticles (NPs), nanowires and nanotubes (NTs) of metal oxides^2^, porous matrices formed by conducting polymers^3^, carbon nanowalls^4^, and nanostructures exhibiting a core-shell^5^ nature where one material is covered by the another. Significant efforts are being aimed especially at the fabrication of those structures where two materials are combined and a new, synergistic effect is observed which is atypical for the single elements^6^. In such heterojunctions, the unique or much enhanced optical, magnetic, electrochemical and sensing properties can arise compared to those exhibited by the individual components. Following this path, heterostructures are formed at least by two different materials. Titanium dioxide nanotubes^7^ are very often used as a highly ordered platform to deposit the other part of the junction, e.g. nanoparticles^8^, quantum dots^9^, electroactive polymers^10^ (polyaniline, polyethylene dioxythiophene) or thin films of different metal oxides^11^ (Fe_2_O_3_, WO_3_). This kind of material composed of tubes placed perpendicularly to the conducting substrate offers their hollow interiors for further modification, and even the space between the tubes if a laterally spaced arrangement^12^ is obtained. Such a well-organised geometry can be achieved via the anodisation of Ti foil^13^ or just a metallic layer already deposited onto a semitransparent substrate, e.g. fluorine-tin-oxide^14^. A change of the anodisation voltage, electrolyte composition, namely the water and fluorine ion content, and the whole process duration allow the geometric parameters of the tubular structure to be controlled: their length, internal diameter, wall thickness, and even the distance between particular tubes^15^. Since such a wide range of different NT morphologies can be easily provided, titania nanotubes remain a very interesting substrate especially once decorated with noble^16^ and non-noble metal^17^ nanoparticles in just the surface region or uniformly, along the whole tube length. Apart from the unique morphology, nanotubes, as one of many various titania nanoforms, also exhibit chemical inertness, high stability, and photocorrosion resistance^18^, which makes them a very promising base targeted to the formation of an attractive heterojunction. In the related literature, numerous approaches were reported on the integration of titania NTs with gold nanostructures, since tiny Au nanoparticles exhibit a plasmon resonance band in the visible range^19^ positively affecting the photocatalytic properties as well as improving the conversion efficiency of solar radiation to electricity^20^. In order to combine the titania NTs with gold species, photocatalytic reduction with the use of a Au precursor^21^, thermal evaporation^22^, electrochemical deposition^23^ and magnetron sputtering without^24^ or with further thermal dewetting^25^ were applied. The latter route can be regarded as a green one because of the absence of by-products and the lack of purification steps required since only a pure gold target is used as the metal source. Notably, the thickness of the deposited Au films and their annealing conditions are considered to be the main factors affecting the geometry and physico-chemical properties of the obtained heterostructure. The annealing process responsible for the formation of NPs from the gold film can be realised under continuous or pulsed mode using furnace or laser radiation, respectively.

Typically, continuous mode is utilised to obtain metallic nanoparticles on a titania support^25^. Two different configurations may be observed for such heterostructures, namely NPs in the crown position (on the rims of the tops of the nanotubes) or after prolonged annealing in the bottom position—i.e. inside of the NTs. Nevertheless, it has already been proven that the Au–TiO_2_NTs junction possesses better photoactivity when Au is only placed on the topmost part of the NTs^25^. As thermal treatment realised in continuous mode—typically in a furnace—is time-consuming and also includes heating and cooling periods, laser irradiation may be used to form the nanoparticles out of thin film. Usually, such processing is used for flat substrates^26,27^ and, to the best of our knowledge, has not been applied until now to fabricate Au–TiO_2_ materials of a high degree of ordering. Apart from the fact that laser treatment is much faster than furnace treatment, also it allows for modification of the sample in the specified area of any shape if template masks are used. Moreover, laser technology has already found numerous applications in industry, what suggests that laser-supported fabrication routes could be easily scaled up from the laboratory to the commercial level.

In this work, we examine the morphological, structural, optical, electrochemical and photoelectrochemical properties of laser-modified Au–TiO_2_NTs. The titania support was obtained via an anodisation process while the Au was deposited by means of a magnetron sputtering system. UV laser irradiation was applied to form metallic nanoparticles, and SEM imaging verified the morphology of the as-prepared materials. Raman, X-ray diffractometric and UV–Vis measurements were performed to study the structure and optical behaviour, respectively. Finally, electrochemical tests were conducted to establish the material’s photoresponse based on the difference in activity when the sample was kept in the dark versus exposed to UV–Vis and visible illumination. Additionally, the concentration of donor density and flat-band potential were estimated from the Mott–Schottky relation. The obtained results enable nomination of the fabricated electrode material with enhanced photoactivity towards successful water splitting process.

## Experimental

The electrode material was fabricated via the following steps: cleaning, anodisation and calcination of the Ti foil, deposition of the gold layer, and finally treatment by laser radiation. Firstly, the Ti foil (Strem, 99.7%) was degreased using acetone, ethanol and water. This process was performed for 10 min for each solvent and the ultrasounds supported the insensitivity of the cleaning. Anodisation was carried out in a two-electrode set up were the Ti plate acted as the anode and a Pt mesh as the cathode. Both electrodes were kept at a distance of 2 cm and immersed in an electrolyte composed of ethylene glycol (85 vol%)/deionised water (15 vol%) with dissolved 0.27 M NH_4_F. The electrolyte temperature was controlled by a thermostat (Julabo F-12) and maintained at 23 °C. The electrochemical oxidation was carried out at 40 V for 15 min. Afterwards, the Ti plate was rinsed in ethanol and calcined in a tube furnace (Nabertherm) for 2 h at 450 °C. The heating rate was set to 2°/min while the cooling to room temperature was performed naturally. Such thermal treatment ensures phase conversion from amorphous to crystalline. Then, 5- and 10-nm thin gold layers were deposited using a magnetron sputtering system (Q150 TS, Quorum Technologies, target: 99.99% Au plate). The bare, unmodified material was labelled as T while those with the gold coating were labelled as TiAu5 and TiAu10, depending on the Au thickness.

Thermal processing of the as-deposited gold layers was realised by utilising a pulsed Nd:YAG laser (Lotis) generating a 355 nm wavelength with a pulse duration of 12 ns, additionally equipped with a laser beam homogeniser to ensure uniform distribution of energy within the spot. The pulse repetition rate was set to 2 Hz and the pulse number to 10. The energy fluence was varied in the range between 30 and 240 mJ/cm^2^. The whole processing took place in air at ambient temperature. For reference, bare titanium dioxide nanotubes also underwent laser treatment. With respect to the applied energy fluence, an additional part was appended to the sample’s name: F30–F240.

**Scheme 1:**
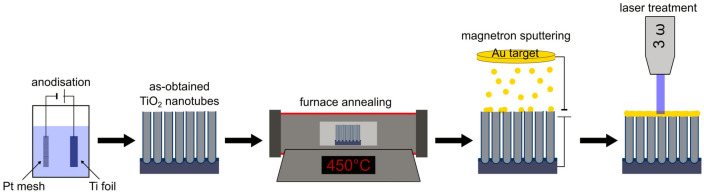
Schematic fabrication process of Au-decorated titania nanotubes—from anodisation to laser processing. Inkscape software was used to prepare scheme.

A representation of the implemented fabrication route is given in Scheme 1.

For comparison purposes, thermal treatment was also performed under continuous mode in a furnace (Neotherm). Two treatment periods were established, namely 10 and 30 min, with the temperature set to 450 °C, and the samples underwent rapid annealing. In accordance with the 10-min or 30-min duration of the furnace processing, an additional segment of 10 m or 30 m was added to the sample name, respectively.

The surface morphology of the titania NTs was revealed using Schottky field emission scanning electron microscopy (SEM, FEI Quanta FEG 250) with an ET secondary electron detector. The voltage was kept at 10 kV.

The structural analysis was carried out by means of a Raman spectrometer (Renishaw In-via) with laser excitation at 514 nm and the laser power reduced to 5 mW, and an X-ray diffractometer (XRD, Bruker D2Phaser) with CuKα radiation equipped with an XE-T detector. In the case of the Raman spectra, the baselines were not corrected.

Optical characterisation of the obtained materials was realised with a UV–Vis spectrophotometer (PerkinElmer) in the range of 280–1100 nm. The scanning speed was set to 60 nm/min. Bandgap energy values were determined as the intercept of the tangent of the plot of transformation of the Kubelka–Munk function.

The electrochemical investigations were performed in a three-electrode arrangement, where the fabricated materials served as the working electrode, a Pt mesh was applied as the counter electrode, while Ag/AgCl/0.1 M KCl was used as the reference electrode. As the electrolyte, 0.5 M NaOH solution, previously purged with Argon 5.0 to remove the dissolved oxygen, was used. During the measurements, an Argon cushion was kept above the solution to prevent oxygen penetration from the environment to the electrochemical cell. The verification of electrochemical activity was carried out based on the recorded cyclic voltammetry (CV) and linear voltammetry (LV) curves as well as a Mott–Schottky (M–S) plot using an Autolab PGStat 302N potentiostat–galvanostat system. The CV scans were recorded in the range of − 0.8 and + 0.8 V versus Ag/AgCl/0.1 M KCl with a scan rate of 50 mV/s and the LV curves were registered from − 1.1 to + 1.5 V versus Ag/AgCl/0.1 M KCl with a scan rate of 10 mV/s. The current density values were calculated using the geometric surface area. The LV measurements were performed in three different conditions: in the dark, under Vis, and under UV–Vis irradiation of the working electrode. To make these tests possible, the electrochemical cell contained flat quartz glass of a size enabling uniform irradiation of the titania NTs plate. As the light source, a stable xenon lamp (Oriel 150 W) equipped with an AM1.5 filter was used, and its intensity (100 mW/cm^2^) was calibrated using a reference Si cell (Rera). In order to generate the visible radiation, a UV filter was also mounted (GG 420, Schott). The M–S plot was prepared based on the impedance data (*f* = 1000 Hz) collected at 50 different potential values changing from + 0.8 to − 0.8 V versus Ag/AgCl/0.1 M KCl. The impedance measurements were repeated twice for the various materials selected on the basis of the CV and LV results. Before recording a single impedance, the potential was held to achieve steady-state conditions. The capacitance of the space charge layer (*C*_SC_) was then estimated from the imaginary part (Z_im_) of the measured impedance according to the relation: C_SC_ = 1/(2π*f*Z_im_)^28^. The value of donor density was determined based on the Mott–Schottky relation where the space charge capacitance of the semiconductor is given by^29^:

$$ N_{d} = \left( {\frac{2}{{\varepsilon \varepsilon_{0} e}}} \right)\left[ {\frac{{d(C_{SC}^{ - 2} )}}{dE}} \right]^{ - 1} $$

where ε (38 for anatase^30^)—the dielectric constant of titania, ε_0_ (8.85 × 10^–12^ F/m)—the vacuum permittivity, *e* (1.602 × 10^–19^ C)—the electron charge, and *E*—the applied potential. For the calculations, the geometric surface area and the same dielectric constant for all of the investigated samples were used. The position of the flat-band potential was indicated as the potential where the tangent to the M–S plot intersects with potential axis for *C*_SC_^−2^ = 0.

Applied in here preparation techniques, namely anodisation, magnetron sputtering and laser annealing, are frequently used on the technological scale in many manufacturing processes. The particular parameters of those methods are highly controlled. According to the numerous morphology inspections and structure analysis for series of produced samples as well as registered electrochemical data we can regard obtained material and its properties as reproducible.

## Results and discussion

### Morphology of obtained materials

**Figure 1:**
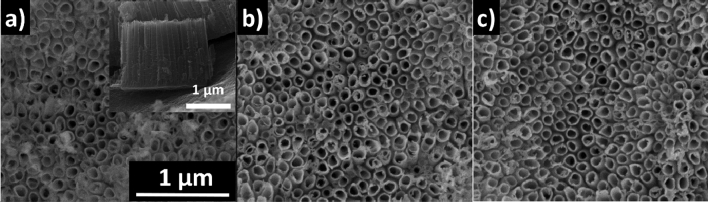
Titanium dioxide nanotubes: bare material (**a**), covered with 5 (**b**) and 10 nm (**c**) Au layers. Inset in (**a**) represents the cross section image of pure TiO_2_NTs.

SEM images of the as-prepared titanium dioxide nanotubes before and after coating with 5 and 10 nm-thick gold films are shown in Fig. 1. As can be observed, the anodisation process led to the formation of a highly ordered titania layer composed of nanotubes of 118 ± 14 nm in diameter, 16 ± 3 nm in wall thickness and 1.48 ± 0.03 µm in length (Fig. S1). The deposition of the thin Au films did not influence the geometry of the TiO_2_NTs and no additional features can be seen for the TAu5 and TAu10 samples.

**Figure 2:**
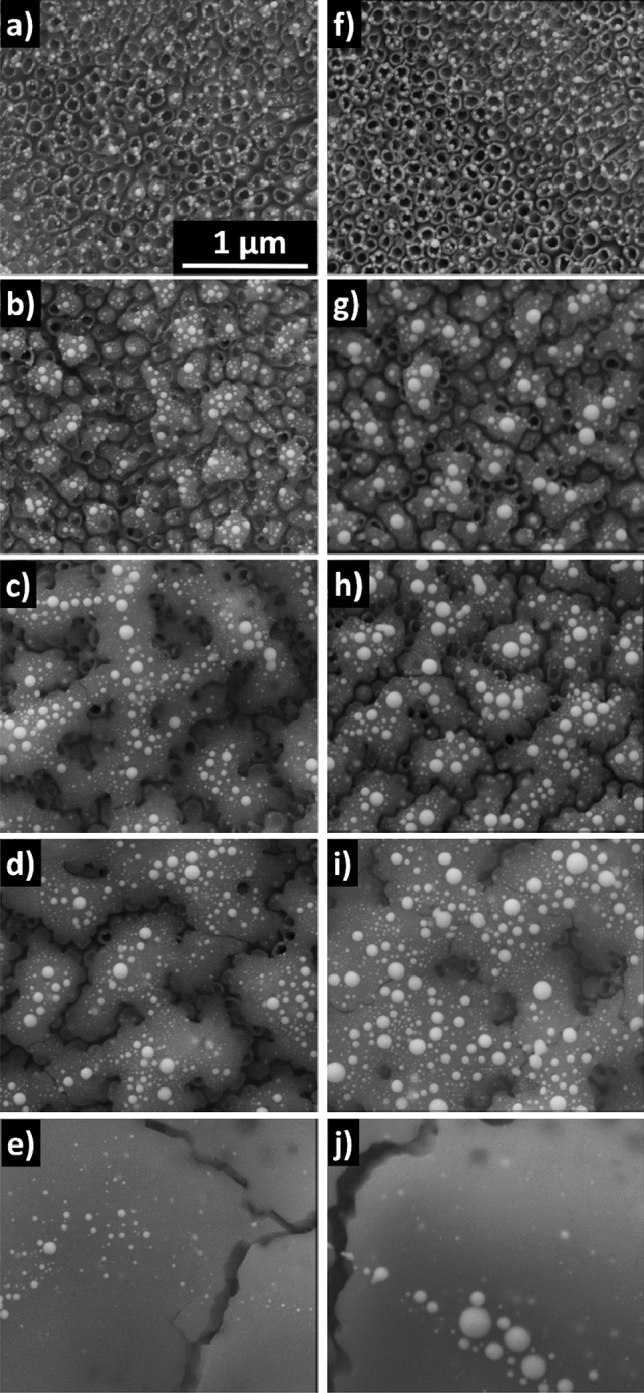
SEM images of laser-treated TiO_2_NTs covered with 5 (**a**–**e**) and 10 (**f**–**g**) nm Au layers with fluences of 30 (**a**, **f**), 60 (**b**, **g**), 120 (**c**, **h**), 180 (**d**, **i**) and 240 (**e**, **j**) mJ/cm^2^.

SEM images of the laser-modified Au-coated TiO_2_NTs are presented in Fig. 2. It can be seen that for both thicknesses of the gold layer, the lowest energy fluence, i.e. 30 mJ/cm^2^, caused the formation of spherical nanoparticles located mostly in the crown position (see also Fig. S2) as was also reported by Nguyen et al.^25^ in the case of furnace dewetted NPs. The position of nanoparticles after dewetting depends on the initial thickness of the gold layer and the sputtering angle. For thin films and shallow angles, the metal coats the tops of the nanotubes and therefore the nanoparticles are formed in the crown position. The influence of metal thickness on the final morphology was also reported by Giermann and Thompson^31^. It was pointed out that the geometry of the substrates is of key importance as well. Hong et al.^32^ provided a similar statement that the geometry of the nanotubes dictates the nanoparticles’ arrangements. Overall, the used parameters (i.e. film thickness and substrate architecture) allowed us to obtain nanoparticles in the crown position. It should be also mentioned that no melting of the nanotubes is observed (see also Fig. S3). However, increasing the laser fluence results in the partial surface melting of the titania tubes and the area of melted regions grows with the fluence up to 180 mJ/cm^2^, while simultaneously, the nanoparticles are also formed. It should be also underlined that beneath the melted layer, the initial architecture of the TiO_2_NTs is preserved (Fig. S4). When 240 mJ/cm^2^ fluence is applied, the melted region completely covers the top of the nanotubes. Moreover, some cracks in the melted layer are visible, as was also observed in the work of Enachi et al.^33^ The same observations, i.e. melting of the outer parts of the tubes while maintaining their initial architecture, can also be deduced for bare TiO_2_NTs treated with laser irradiation—see Fig. S5 in the Supplementary Information file.

As it comes to the size of formed nanoparticles, it can be seen that it depends for the smallest fluence on the film thickness and the average size and the standard deviation rise after applying higher fluence (Fig. S6). The dependence of the NPs size on layer thickness is in agreement with literature data^34^. For higher fluences the average size of nanoparticles is independent on applied energy what is consistent with the works of Ratautas et al.^35,36^. The only exception is for sample covered with 10 nm thick Au layer and processed with 240 mJ/cm^2^ fluence. This may be caused by the changing of morphology of the substrate due to laser treatment as for this fluence the melted layer completely covers the top of the nanotubes. It is also observed that the nanoparticles number is decreasing with the laser fluence. Nevertheless, it should be also kept in mind that the laser processing of metallic films leads to the formation of nanoparticles exhibiting quite broad size distribution and during multipulse irradiation two main competitive processes occur influencing the size of nanoparticles: merging and decomposition of formed NPs^27^.

As was mentioned in the Experimental section, for comparison reasons, nanotubes coated with Au layers underwent thermal treatment in continuous mode in two different time regimes. This processing, as expected^25^, led to the fabrication of Au nanoparticles located in the crown position. The illustrating SEM images can be seen in Figs. S2 and S7. In this case, also the size of nanoparticles depends on the initial thickness of Au layer (Fig. S8), however standard deviation is much smaller. Moreover, the nanoparticles diameter is not changing in a significant way with the treatment duration. The values of average size of nanoparticles and standard deviations are given in Figs. S6 and S8.

### Structural properties

**Figure 3:**
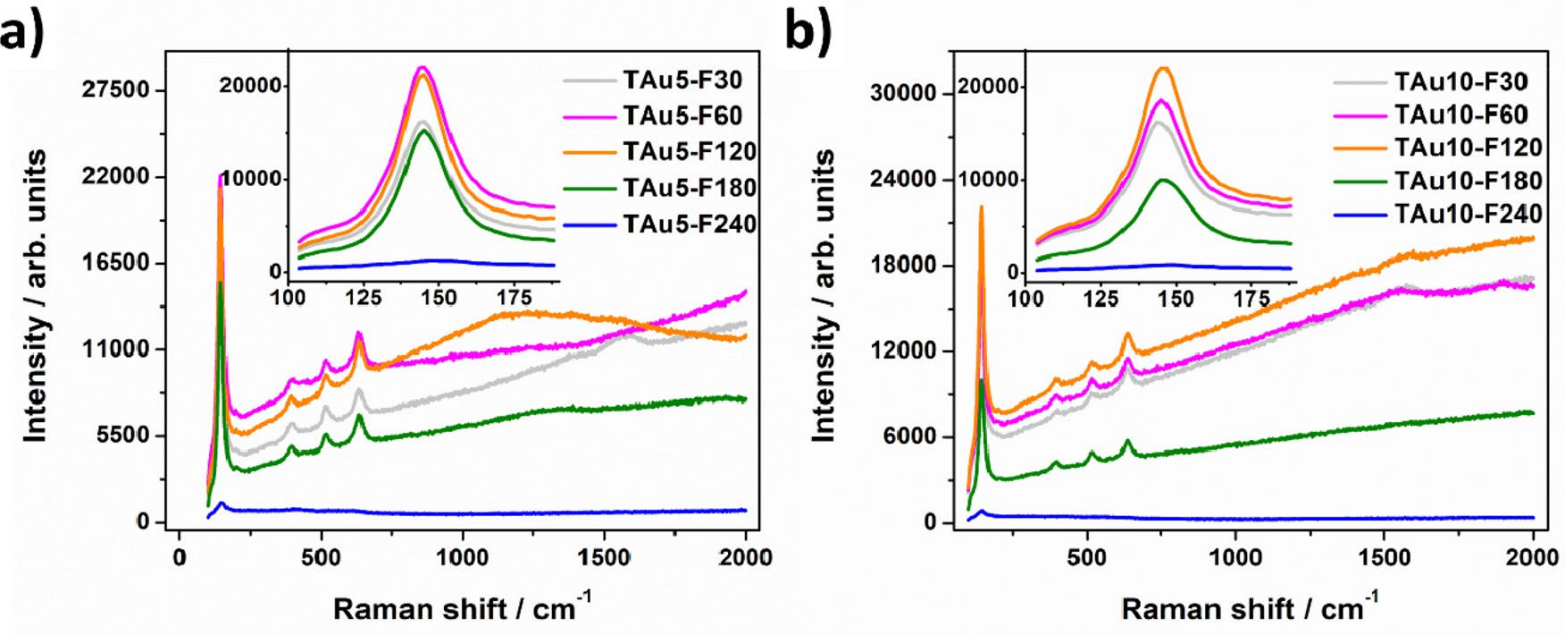
Raman spectra for laser-treated samples covered with 5 (**a**) and 10 (**b**) nm Au layers.

To study the structural properties of laser-modified Au–TiO_2_NTs, Raman measurements were carried out for all prepared samples. It can be seen in Fig. S9 that pure titanium dioxide nanotubes exhibit an anatase crystalline phase, as characteristic peaks are located at: 144, 197, 396, 515 and 638 cm^−1^. They are related to the following modes: E_g(1)_, E_g(2)_, B_1g_, A_1g_ and E_g(3)_^37^_,_ respectively. After the deposition of the gold layers and thermal treatment in the furnace, no additional peaks arise and no shift of peaks is observed (see Figs. S10 and S11) meaning that no embedding of metal into the titania structure occurs. For the laser-treated Au-coated samples, also no change in peak position can be seen when irradiation of fluence below 240 mJ/cm^2^ is applied—Fig. 3. Moreover, the crystal structure and anatase phase is preserved. Consistently with the work of Zhang et al.^38^ for increasing temperature (in our case, increasing laser fluence up to 120 mJ/cm^2^), the enhancement of the Raman signal accompanied with the narrowing of the E_g(1)_ band is observed. However, for a fluence of 180 mJ/cm^2^, the intensity of the Raman signal decreases, which may be attributed to the geometry change within the tubular structure. When 240 mJ/cm^2^ fluence is used for the laser processing, almost no peaks are visible, indicating possible degradation of the crystal structure in the topmost zone of the sample.

Interestingly, if pure TiO_2_NTs are irradiated by laser, initially the Raman signal rises and no shift is observed for the main active anatase mode—see Fig. S12. However, for a fluence of 120 mJ/cm^2^ and higher, the peak intensity decreases while its position is shifted to higher frequencies (~ 149 cm^−1^) which can be related to the formation of oxygen vacancies during the process^39^. The used laser wavelength corresponds to the bandgap energy of titania, therefore laser irradiation excites the electrons from the valence band (VB) to the conduction band (CB). Afterwards, a hole is captured by an electron from an adsorbed oxygen species (O^2−^/O_2_^2−^ level) while a photoelectron from the CB is trapped in Ti^4+^ forming Ti^3+^—an oxygen vacancy. The presence of adsorbed reactive oxygen species is highly probable since the laser modifications were performed under ambient air. The proposed mechanism is described e.g. by Lau et al.^40^ and Ricci et al.^41^. Going forward, as in the case of Au-covered titania material, for a 240 mJ/cm^2^ fluence, the degradation of the crystal structure is observed most likely due to heat accumulation at the top of the nanotubes. These results indicate that the additional gold layer may protect the material from the formation of oxygen vacancies with simultaneous distortion of the crystal structure.

Additionally, XRD measurements were conducted (Figs. S13–S16). For bare titania, the detected XRD patterns can be mainly assigned to anatase (25.26°, 48.01° and 55.13°, PDF-2 01-071-1168) and titanium (35.09°, 38.43°, 40.19°, 52.98°, 63.01°, 70.67° and 76.29°, PDF-2 00-044-1294). Nevertheless, a small signal from rutile is also observed (27.44° and 54.16°, PDF-2 01-076-0320). After deposition of the gold layers, a small peak at 44.40° of the gold phase (PDF 2 00-001-1172) appears for the 10 nm layer. The intensity of this maximum rises with the increase in laser fluence (Fig. S16), which is consistent with the SEM observations, which show a growth in the size of gold nanoparticles on the surface. These effects are barely visible for the 5 nm gold layer due to the resolution of the method. When pristine titania is processed by means of UV-laser irradiation, the ratio of anatase to titanium is decreased for the highest fluences, which is consistent with the Raman results where a lowering of the main anatase peak is observed with the increasing laser energy. Moreover, no rise of peaks corresponding to rutile is noted. In addition, a reduction in the ratio of the Ti (103) peak—2Θ = 70.67° to the Ti (002)—peak 2Θ = 38.43°, is noted. The titanium plane (103) is dominant for the structure of the nanotubes, so, as expected, the higher laser energy shortens the length of the nanotubes and, consequently, their signal content is smaller. A similar trend is exhibited by the laser-treated samples covered with 5 and 10 nm of Au. Nevertheless, we can assume, taking into account the fact that the chosen fluences are below the ablation threshold (meaning that no evaporation of material should occur), that the laser processing may lead to some degradation of the crystal structure. The possible formation of local amorphous phases may occur, as during re-solidification, there is not enough time for recrystallisation due to the laser-processing timescale. That is evidenced by both the XRD (anatase-to-titanium ratio) and Raman (lowering of the peak assigned to E_g(1)_) data. Moreover, the modification of the pristine titania, namely the deposition of the Au layer, as well as thermal treatment in continuous and pulsed mode, leads to the XRD peak shift (Figs. S17–S20). Such a shift may be an indicator of oxygen vacancy formation for laser-treated samples. It should be underlined that no trend in peak shifting is seen for the particular processing parameters. Nevertheless, it depends on the modification conditions^42^ and since laser treatment is a nonlinear process, such an observation can be expected.

### Optical properties

In order to study the optical properties of the prepared materials, the reflectance spectra were measured, and are shown for all of the samples in Figs. S21, S22 and S23. The spectra for pure titanium dioxide nanotubes are characteristic for this kind of material, and were previously reported in our other work^43^. It should be remembered that the transition of an electron from the valence to conduction band is responsible for the large absorption in the UV region. Moreover, the increased absorption from 450 up to 1100 nm can be ascribed to the scattering of light by pores or cracks in the titania arrays^44^. The deposition of gold layers of both used thicknesses led to the appearance of slightly visible fringes. This can be explained by: surface plasmon resonance (SPR) as both the 5 and 10 nm-thick films can be considered as non-continuous, the photonic behaviour of the material, or constructive and destructive interference of reflected light^45^. Nevertheless, after thermal treatment, a new absorption band can be distinguished in the visible region with a maximum at ca. 580 nm. This can be related to the formation of Au nanoparticles out of thin layers, and the occurrence of SPR. A similar behaviour can be observed for the laser-treated samples previously coated with metallic films. However, in this case, the plasmonic bands exhibit much lower intensity and increased reflectance in the UV region is observed for higher energy fluences, especially when the 10 nm Au film was formerly deposited onto the TiO_2_NTs. This can be ascribed to the formation of melted fragments clearly visible in the SEM images. Meanwhile, for the laser-treated bare titania nanotubes, the absorption of materials decreases with the increased energy fluence, which is consistent with the observations made on the basis of the SEM analysis. It is already established that with the growth of the laser energy, the melted area enlarges and therefore the real surface area of the material decreases. The observed increase of reflectance with the lowering of the surface area is in agreement with the works of other researchers^46^.

**Figure 4:**
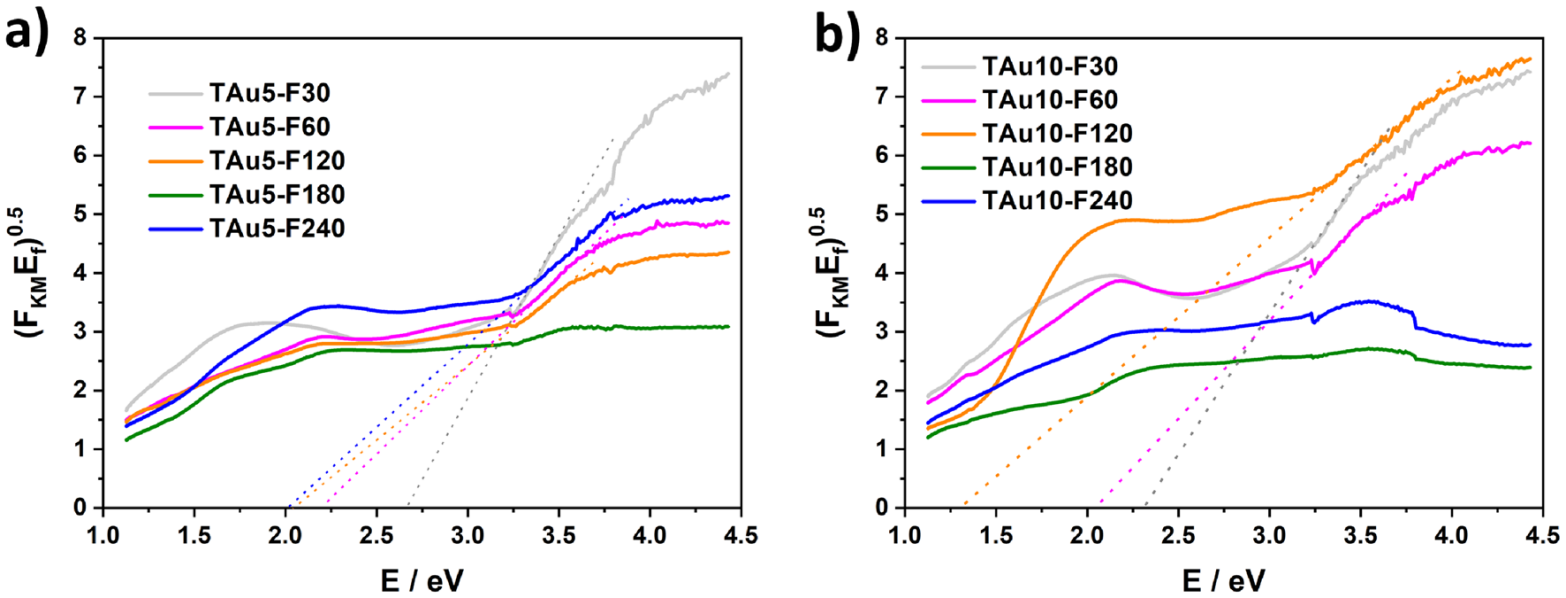
Tauc plots of laser-treated TiO_2_NTs covered with 5 (**a**) and 10 (**b**) nm Au layers.

The influence of laser processing on the bandgap value (E_bg_) is examined on the basis of Tauc plots prepared by using the reflectance spectra and the Kubelka–Munk relation—see Figs. S21, S22 and Fig. 4. For clarity, all of the values are presented in Table 1. The E_bg_ for pristine titania nanotubes equals 2.9 eV, which is narrower than the one reported for bulk anatase—3.2 eV. Nevertheless, one should take into account that the energy bandgap strongly depends on the geometry of the studied material. It has also been proven that in the case of TiO_2_NTs, E_bg_ changes are related to the wall thickness, diameter and length of the tubes^47^. After deposition of gold and further treatment of the samples in the furnace, the narrowing of E_bg_ is seen. The narrowing of the bandgap may be attributed, e.g., to the introduction of the additional states (“shallow donors”) resulting from the degradation of the crystal structure of the TiO_2_^48^, the formation of oxygen vacancies, titanium vacancies or oxygen interstitial defects^49^, or even the introduction of the intra-gap energy due to the interaction of Au and TiO_2_^50^. In the case of furnace-treated samples the variation of E_bg_ can be ascribed to the interaction of Au and TiO_2_.

**Table 1:** Energy bandgap values (E_bg_/eV) for all prepared materials.

Sample	E_bg_	Sample	E_bg_	Sample	E_bg_	Sample	E_bg_
T	2.9			TAu5	2.74	TAu10	2.45
T-F30	2.93	TAu5-10m	2.72	TAu5-F30	2.66	TAu10-F30	2.31
T-F60	2.96	TAu5-30m	2.73	TAu5-F60	2.21	TAu10-F60	2.04
T-F120	2.97	TAu10-10m	2.46	TAu5-F120	2.04	TAu10-F120	1.3
T-F180	2.24	TAu10-30m	2.53	TAu5-F180	–	TAu10-F180	–
T-F240	2.91			TAu5-F240	2.02	TAu10-F240	–

Laser treatment of coated titania nanotubes led to the further decreasing of the energy bandgap values and the lowest value is estimated for the TAu-F120 sample. As laser-processed samples possess lower values of energy bandgap than furnace treated ones, the changes of E_bg_ may be caused by two combined effects, namely the presence of gold and the introduction of oxygen vacancies, which is supported by the XRD and Raman data. Nevertheless, for the TAu5-F180, TAu10-F180 and TAu10-F240 samples, it was impossible to estimate E_bg_ as the absorption band edges could not be distinguished. This may be related to the oxygen deficiency and thus the presence of Ti^3+^ ions^46^. Interestingly, the values for the laser-irradiated pristine titania materials barely change in comparison to the unmodified sample, with the exception of TiO_2_NTs treated with 180 mJ/cm^2^. This can be attributed to the fact that the melted region of the titania surface still possesses the anatase crystalline phase and therefore that the presence of Au is responsible for the different behaviour of the metal-covered nanotubes.

### Electrochemical activity

**Figure 5:**
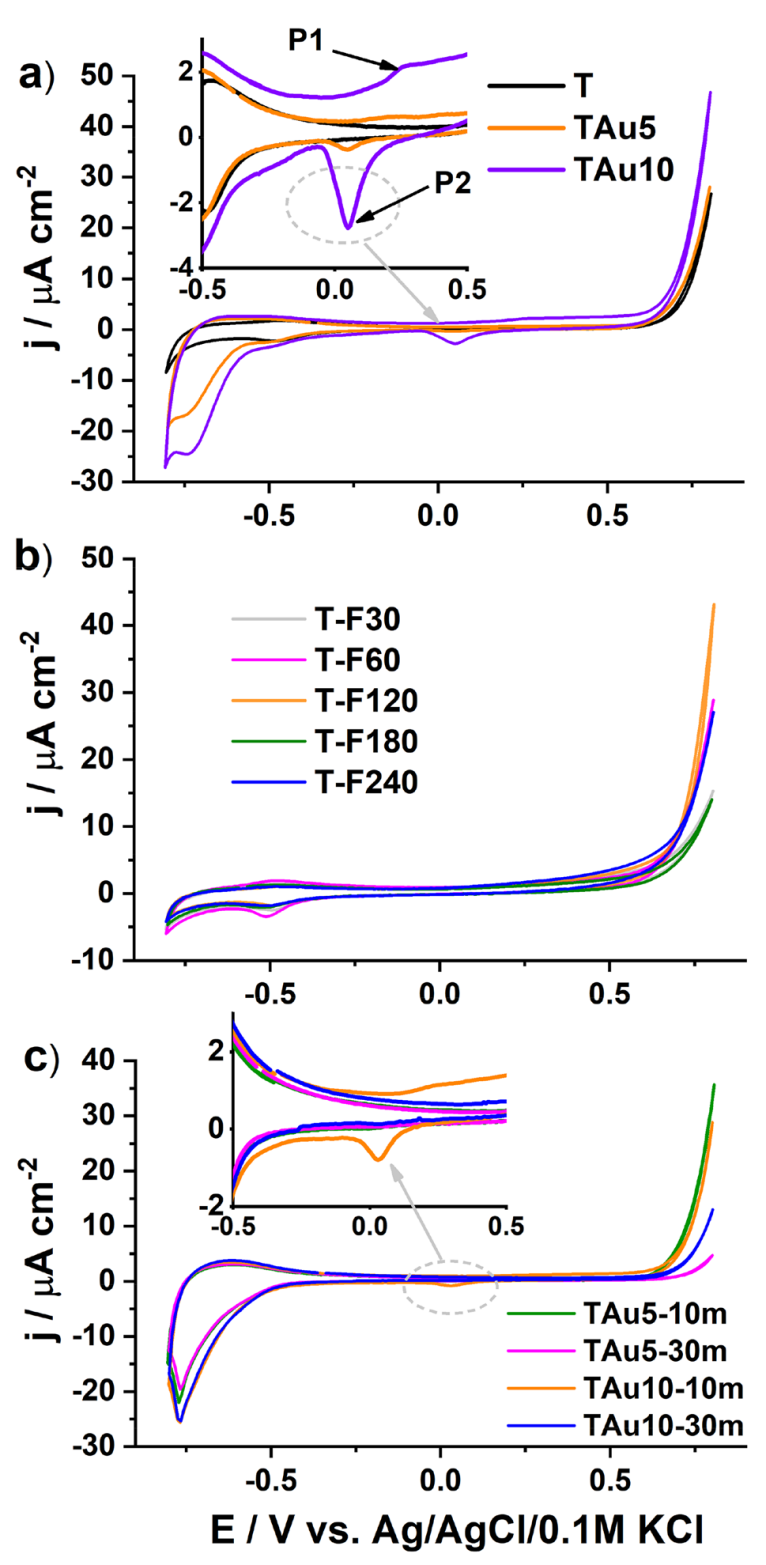
Cyclic voltammograms recorded for the sets of reference materials: (**a**) bare and gold sputtered titania, (**b**) laser-treated bare titania nanotubes, (**c**) titania with deposited 5 and 10 nm gold films and thermally treated in a furnace.

In order to show the extraordinary properties of the fabricated samples, at first, electrochemical studies were performed for the set of reference materials, see Fig. 5. In Fig. 5a, the CV recorded for the bare sample and the titania with the sputtered thin gold layer are shown. The shape of the TiO_2_NTs exhibits a very low capacitive current and only a small peak is present at − 0.5 V versus Ag/AgCl/0.1 M KCl, which corresponds to Ti(IV)-to-Ti(III) reduction^51,52^. When the Au coating is present, both in the anodic and cathodic regimes, higher currents were recorded, while for the TiAu10, an additional reduction peak even arises at + 0.05 V and an oxidation current with a small peak at + 0.2 V can be found. This P1 signal is attributed to the formation of a gold oxide surface monolayer, while on the reverse scan, P2 results from the reduction of gold oxides^53,54^. Sometimes before oxidation, a small wave is observed due to the partial charge transfer of the chemisorbed OH^−^ anions and peroxidation of the gold surface^55^, but here, such behaviour cannot be distinguished. In the cathodic limit, much higher current densities compared to the bare titania are also observed, and are assigned to the hydrogen evolution. Part b) of Fig. 5 presents the CV for the titania treated only with the laser beam of various fluences. Comparing the shapes of the CV curves recorded for the set of TiO_2_, one may observe only a difference in the anodic regime, but in the range from − 0.8 to + 0.5 V, the CV almost overlaps. Therefore, laser annealing of pure TiO_2_NTs does not change their electrochemical properties in any significant way.

Some of the increased current density for the laser treated titania results from the higher donor density, as has already been reported by Haryński et al.^56^ and Xu et al.^48^. Similar behaviour was observed by other researchers^57^ and justified by the higher electron mobility, as expected for high-aspect-ratio materials. For the last set of reference materials with the CV given in Fig. 5c, namely those annealed traditionally in the furnace, the electrochemical activity was nearly the same as for the titania (TiAu5 and TiAu10) before such a treatment, as presented in Fig. 5a. Only in the case of TAu10-10m, the signal originating from the reduction of gold oxides remains. Nevertheless, in the investigated potential range, no significant increment of current density in either the anodic or cathodic limit is observed. The value of j due to any of applied modification does not exceed 45 µA/cm^2^ and − 35 µA/cm^2^ at + 0.0 and—0.8 V versus Ag/AgCl/0.1 M KCl, respectively. Moreover, comparing all of the gathered CV curves shown in Fig. 5, one may observe some shift in the initiation of the water oxidation in the anodic regime. As is known, the process of water molecule splitting occurs at the electrode surface starting from the water molecule adsorption, and thus the surface conditions here play a very important role. Since the proposed modification affects the topmost zone, as confirmed by SEM inspection, the presence of a thin gold film before and after furnace treatment (TAu10 and TAu5-10m, respectively) as well as the remelted titania top (T-F240) results in some lowering of the water oxidation potential. Such changes in the behaviour of titania were also observed by Pei et al.^58^, indicating that the defective oxygen vacancies improve the catalytic properties while the gold nanostructures act as reactive centres for further oxygen evolution^59^.

Summarising the performance of the reference materials, one can conclude that the simple deposition of a thin gold coating as well as annealing in a furnace do not lead to any impressive improvement of the electrochemical properties which are worth further investigation.

**Figure 6:**
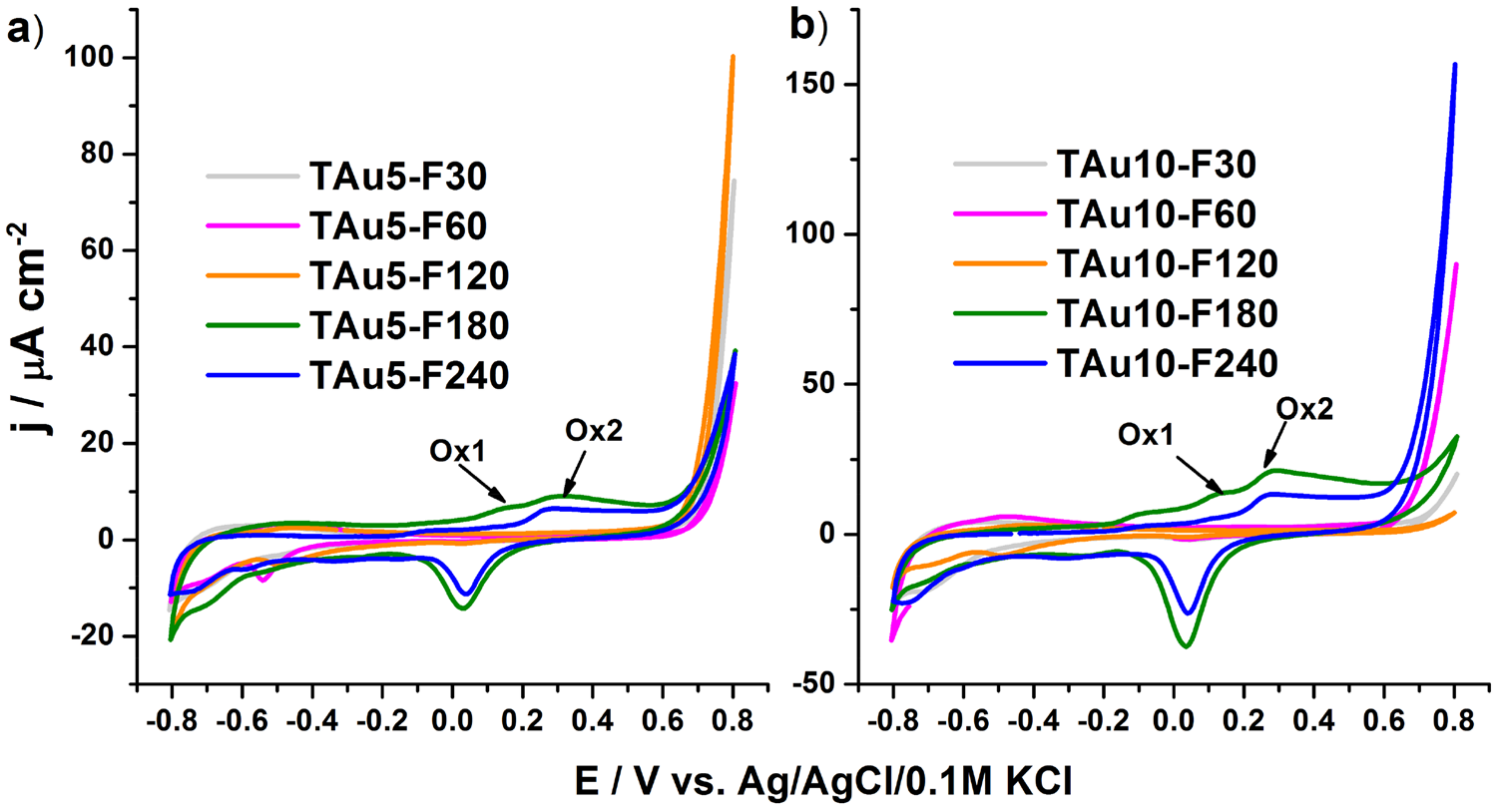
Cyclic voltammetry curves registered for the titania NTs coated by (**a**) 5 nm and (**b**) 10 nm of gold and irradiated by laser with various fluences ranging from 30 to 240 mJ/cm^2^.

Contrary to the reference materials, the treatment with monochromatic intense pulsed light results in electrode material of completely different features. In Fig. 6, the cyclic voltammetry curves are presented for the titania on which gold films were deposited and then laser annealing was carried out. Those materials exhibit highly improved activity in the anodic regime while the signals typical for the formation of gold oxides and their reductions are enhanced. However, the highest increment can be seen above + 0.6 V versus Ag/AgCl/0.1 M KCl and reaches even 150 μA/cm^2^, while for the reference material, the current density does not exceed 50 μA/cm^2^. Moreover, it should also be pointed out that on the anodic CV branch, for the electrodes treated with 180 and 240 mJ/cm^2^ fluences, two oxidation signals can be distinguished, one at + 0.13 V (Ox1) and the second at 0.3 V (Ox2). A similar set of oxidation peaks were reported by Tang et al.^60^ for octahedral Au nanoparticles and justified by the facet-dependent features^61^. Therefore, the interaction between the laser light and gold-coated titania does not affect changes only within surface morphology, but also implies an electrochemical response. Such features exhibited in dark conditions can be used towards the detection of a particular analyte e.g. glucose. In the case of glucose, the detection is based on glucose-to-gluconolactone oxidation, which is greatly dependent on the amount of the AuOH sites which are considered as active species, crucial for the operation of non-enzymatic sensors^62^. Moreover, gold nanostructures can be further modified via, e.g., anchoring of DNA strains^63^ or adsorption of proteins^64^, which then act as highly selective sensing platforms. Taking into account the promising oxidation behaviour observed in the anodic limit, additional linear voltammetry studies were carried out.

**Figure 7:**
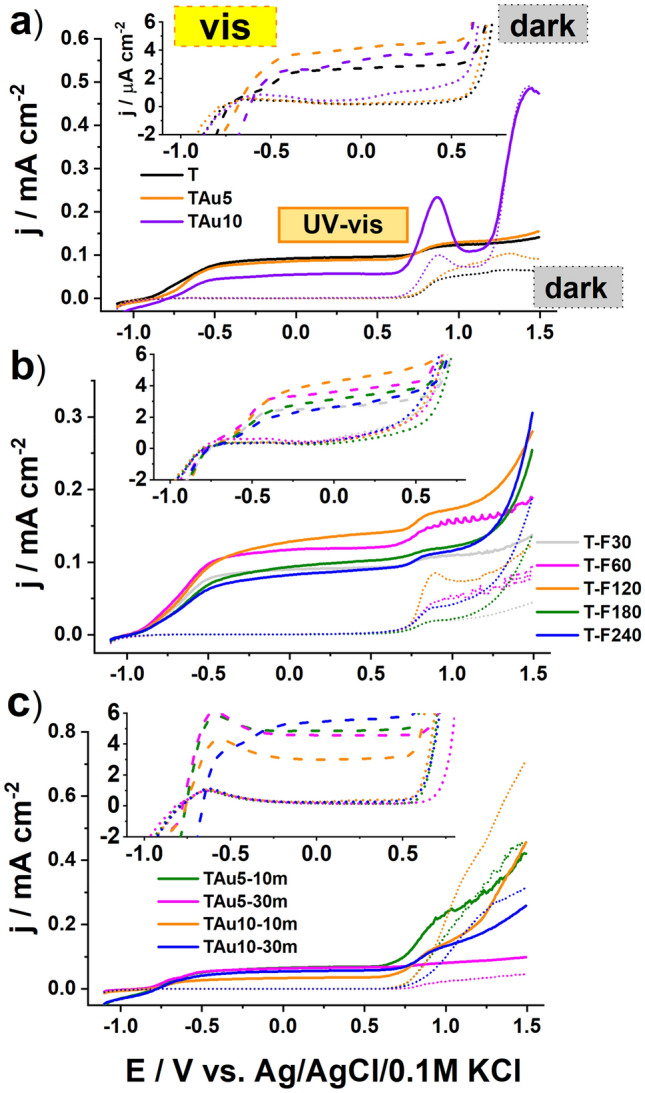
Linear voltammetry curves recorder for different reference materials: (**a**) bare titania and covered by 5 and 10 nm of thin gold layer, (**b**) bare titania treated by laser radiation with various fluences, (**c**) titania NTs coated by gold and thermally treated in a furnace.

As was in the case with the CV, the set of LV measurements were performed first for the reference samples in three different conditions: in the dark, under vis, and under UV–Vis illumination generated by a solar simulator with and without a UV cut-off filter, see Fig. 7. For all of the reference materials, the activity exhibited under visible light is very low and the current density at the plateau maintained until + 0.5 V equals only ca. 4 μA/cm^2^. Such meagre photoactivity results from the wide bandgap energy (fitting the range 2.9–3.0 V) and thus the utilisation of most of the solar spectrum is hampered. When the higher anodic polarisation is applied to the working electrode, a rapid current increase is registered, but only for TAu10, T-F240 and TAu10-10m, among the other samples. This activity is directly related to the water oxidation process, but very small gas bubbles at the electrode surface are generated. However, even if + 1.5 V is reached, the current density does not exceed 0.5 mA/cm^2^. Regarding the situation where the photoelectrode is exposed to the whole solar spectrum, the LV shape for some of the reference materials is much more complex. In the case of the pure titania, the current increases because the UV light contributing to the whole solar spectrum is highly absorbed by the material, and in consequence, excitons are formed. After their dissociation, holes take part in the oxidation processes at the electrode/electrolyte interface while electrons are collected at the Ti back contact resulting in the current flow. When the 10-nm-thin gold layer is deposited, apart from the plateau region, an additional oxidation signal found at + 0.9 V and further strong enhancement are observed. This distinguished peak can be ascribed to the formation of oxides whose exact position depends strongly on the size and shape of the gold species^65,66^.

Regarding the bare titania treated with laser, one may observe that the interaction of intense light with titania nanotubes can affect the material’s photoactivity and in the case of 120 mJ/cm^2^, the current density was 40% higher than for the unmodified sample. Such a positive effect is in agreement with our previous work and was explained by the additional shallow states formed due to the laser modification and some distortion of the crystalline structure within the melted surface region^56^. Therefore, despite our experiment being performed in ambient air conditions and the applied laser radiation having a much longer pulse duration, the observed results are similar. Finally, regarding the thermal treatment realised within 10 or 30 min in the furnace, the analysis of the LV scans presented in Fig. 7c indicates that such a standard approach does not provide any material exhibiting extraordinary electrochemical features for further utilisation, and a decrement of the water oxidation process upon UV–Vis light was even noticed. This phenomenon probably results from the hot electron injection from the titania to the formed gold nanoparticles at the NTs rims, and therefore recombination processes with holes accumulating at the electrode/electrolyte interface take place.

**Figure 8:**
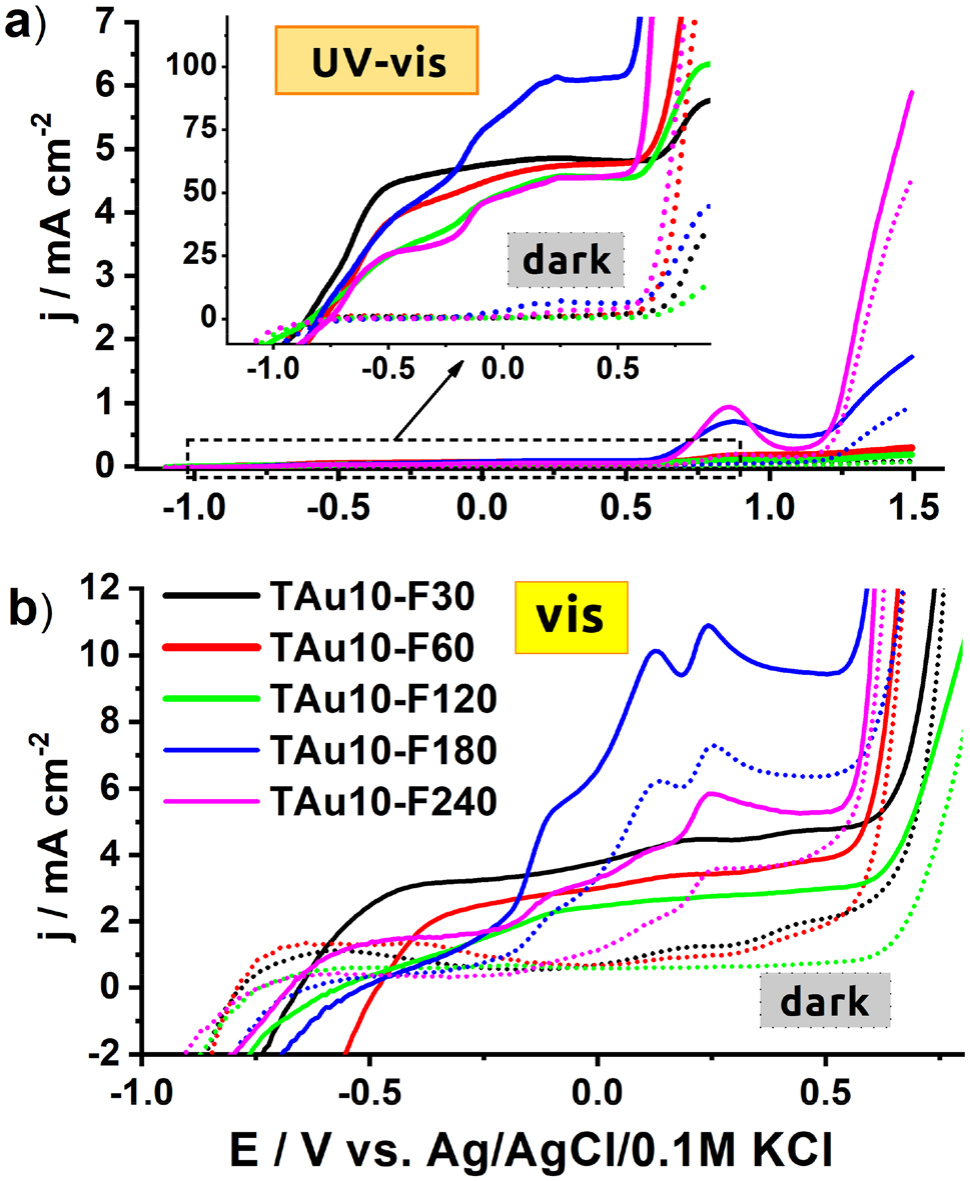
Linear voltammetry curves recorded under (**a**) UV–Vis and (**b**) Vis irradiation for laser-modified samples with initially deposited 10 nm Au film.

Realising that both rapid thermal treatment performed in the furnace or just simple metal layer sputtering meet a threshold regarding the electrochemical response, the unexpected behaviour for the material after its interaction with pulsed laser radiation was found and presented in the Fig. 8 and Fig. S24. When the LV was recorded in the dark, much increased currents in the anodic limit were recorded for the highest energy fluences, namely 180 and 240 mJ/cm^2^. It should be noted that for the highest laser fluence applied towards the titania with deposited 5 nm-thin Au, the current density exceeds 2 mA/cm^2^, while for the 10 nm gold coating, the current density noted in the dark was even above 4 mA/cm^2^. This last mentioned value is ca. 8.5 times higher compared to that observed for TiAu10, which indicates the significance of the changes in the material photoactivity induced by the laser interaction.

When the experiment was carried out under the whole solar spectrum and we tracked the changes of the LV curve, the following features can be described: the onset of the current increase is observed at ca. − 0.8 V, and then reaches a constant value near − 0.5 V, then, above + 0.5 V, the plateau ends and further rapid increase occurs. Comparing the run of LV curves within the range from − 0.8 to + 0.5 V, in general, the current density for the laser-modified materials is lower compared to the bare titania, and as the irradiation fluence increases, the current response diminishes. However, among other laser-treated samples, for the 180 mJ/cm^2^ fluence, the highest photoresponse in this particular range is reached. Going towards a more anodic direction, for laser treatment at fluencies of 180 and 240 mJ/cm^2^ and at both gold thicknesses, the local current maximum appears at + 0.8 V. It results from the oxidation of surface oxides, which has been already found for the TAu5 and TAu10 materials. When the polarisation is further increased, the current rapidly increases, but significantly higher values are reached compared to the dark conditions, and dynamic generation of gas bubbles at the electrode surface is observed. Therefore, the water decomposition process was enhanced by light radiation especially in the case of materials treated with the highest laser fluences, namely 180 and 240 mJ/cm^2^. For the TAu10-F240 sample, the current density reaches almost 6 mA/cm^2^ while, as has already been mentioned, for the reference materials, the value of *j* at 1.5 V was below 0.5 mA/cm^2^ even when the electrode was exposed to the full solar spectrum. For better clarity in showing the improved activity in the anodic limit, the set of current densities recorded at + 1.5 V versus Ag/AgCl/0.1 M KCl under solar irradiation are listed in Table S1.

The behaviour of laser-treated TiAu electrodes was also verified under λ > 420 nm and is presented in Fig. 8b and S21b. Compared to the results obtained in the dark and under UV–Vis radiation, the exposure of the working electrode annealed by 180 and 240 mJ/cm^2^ fluence to visible light leads to the intensification of electrochemical activity at ca. + 0.13 V and additionally at + 0.25 V, which was not observed for the reference TiAu10 and TiAu5 substrates. In the indicated positions for the TAu10-F180 sample, the current density exceeds 10 μA/cm^2^, while for other materials, *j* is below or nearly 5 μA/cm^2^ when the sample is exposed to the visible radiation. This feature has already been explained by the oxidation of the specific face of gold species formed by laser processing over the titania support. Moreover, the most prominent response may also be related to the optical properties, particularly with the high absorption in the visible range of 400–800 nm (see Fig. S23).

Realising that photostability is other crucial feature of an electrode material exposed to light, the transient currents in different light conditions were recorded. The example of the chronoamperometry curve for the TAu10nm-F240 sample is given in Fig. S25, and is similar for all of the investigated materials, both reference and modified. The run of transient photocurrents indicates that each time the material is illuminated, the current rapidly grows and reaches a plateau, whereas when the access to light is cut off, the current density decreases to the initial dark current value. Such behaviour evidences the low recombination rate and stable working of the material under irradiation, favouring its usage in light-driven processes.

Moreover, we compared our best samples to other reported Au–TiO_2_-based materials prepared using different techniques—see Table 2. As can be seen, the values of the enhancement factors (EF, between Au-decorated and pristine titania) for the prepared materials may be regarded as satisfactory and fully justify the usage of the laser.

**Table 2:** Comparison of performance of Au–TiO_2_ materials.

Material	Method of modification	EF	References
Au/TiO_2_-NTs	Pulse electrodeposition	3.2 (visible)	^67^
Au@TiO_2_	Electrochemical deposition	2.4 (solar)	^68^
		1.75 (UV)	
Au/TiO_2_NTs	Photoelectron-deposition	3 (UV)	^69^
TiO_2_/Au	Electrodeposition	2.33 (UV)	^70^
Au/TiO_2_NRs	Constant current electrodeposition	2.19 (UV)	^71^
Au/TiO_2_NRs	DC sputtering	1.38 (solar)	^72^
		4 (visible)	
Au/TiO_2_NTs	Thermal evaporation	1.9 (visible)	^22^
AuAg@TiO_2_	Sputtering and Ar annealing	1.7 (solar)	^25^
Au/TiO_2_ ARHN	Sputtering and calcination	3 (solar)	^73^
Au/TiO_2_ BNRs	Photoreduction	2 (solar)	^74^
TAu10-F240	Magnetron deposition and laser dewetting	12 (solar)	This work
TAu10-F180		2 (visible)	

*NRs* nanorods, *ARHN* anatase/rutile hierarchical network, *BNRs* branched nanorod arrays.

**Figure 9:**
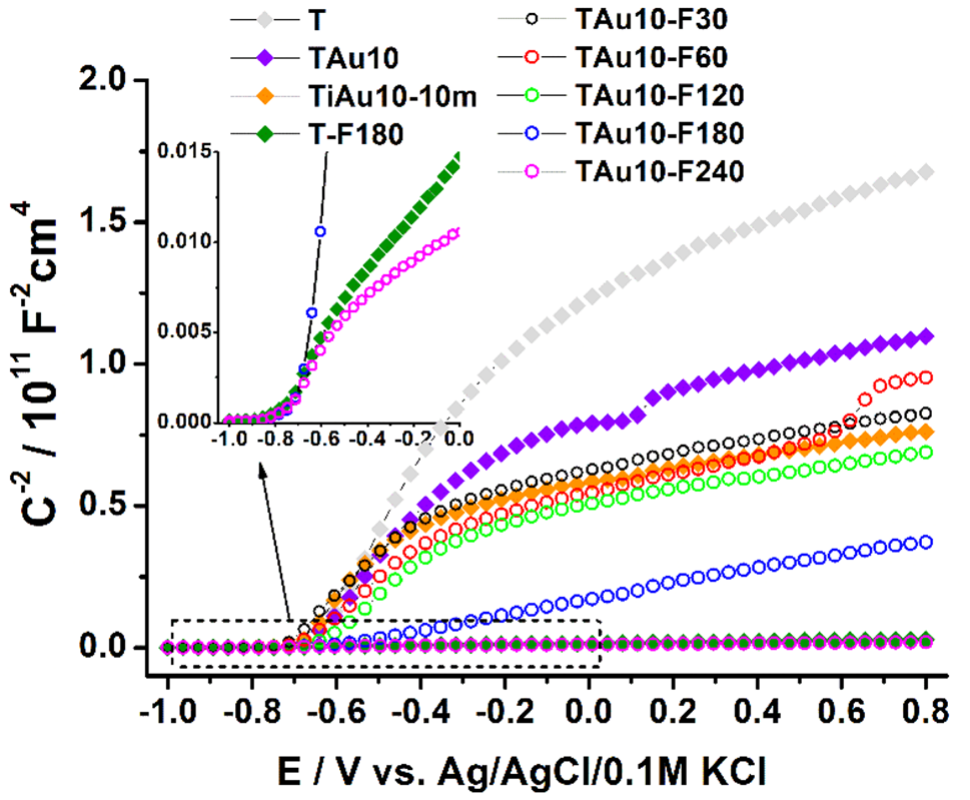
Mott–Schottky plots for the reference and laser-treated materials with an energy fluence of 30–240 mJ/cm^2^.

In order to support the analysis of the synergistic effect between titania with thin gold film and further laser treatment and the resulting photoresponse, Mott–Schottky measurements were carried out. Those studies were performed for the selected set of samples including the reference ones and are presented in Fig. 9. Moreover, based on the run of the M–S plot and the equation provided in the experimental section, the position of the flat-band potential and the values of donor density were determined—see Table 3. First, comparing the position of the flat-band for bare titania before and after laser treatment, one may observe a negative shift of E_fb_ from − 0.67 V (T) to − 0.79 V (T-F180). This change equals only 120 mV, and is not as significant as in the case reported by Haryński et al.^56^. Nevertheless, the experimental conditions applied here, particularly the ambient air and much longer pulse duration, lead to an only slightly higher Schottky barrier at the electrode/electrolyte interface. Analysis of the location of *E*_fb_ for other materials indicates that the values of the flat-band potentials almost overlap and fit the range within − 0.66 V (for TAu10) and − 0.77 V (for TAu10-F240). The opposite situation occurs regarding the change in concentration of donors. For reference, N_d_ for unmodified titania equals 1.61 × 10^19^ cm^−3^, while as the laser fluence increases, the N_d_ value increases even to 1.73 × 10^21^. Thus, a two orders of magnitude difference can be noticed if the laser treatment is applied, while standard thermal annealing barely influences this parameter. A much higher donor density results from the increased oxygen vacancies, which are known to be an electron donor for TiO_2_^75^_._ The enlarged population of electron donors supports charge transportation, and as a consequence, contributes to the overall, enhanced material photoresponse. Additionally, it should be pointed out here that for all of the investigated materials, the same dielectric constant is taken into account and we consider the geometric area not the real one. Those assumptions are typically applied for other modified titania, e.g. with metal and non-metals^75,76^, but here we are dealing with the laser treatment exhibiting a non-linear character and the final effect strongly depends on the initial conditions. More specifically, when the gold film is deposited onto the titania, the laser interaction with the sample surface is different regarding the case of the bare substrate. Because of that, for the nanotubes with the sputtered gold layer, a higher laser fluence is needed to increase the donor density compared to the reference material. It should also be highlighted that a very high donor density does not always lead to prominent photoactivity. In the work of Sun et al.^77^, the reported material exhibiting the highest donor density did not generate the highest photocurrents. It was justified by titania band shifting and the creation of much more interfaces which impede the charge separation.

**Table 3:** The values of flat-band potentials and concentration of donor density estimated based on Mott–Schottky plots (see Fig. 9).

Sample	Flat-band potential V versus Ag/AgCl/0.1 M KCl	N_d_/10^19^ cm^−3^
T	− 0.67	1.6
TAu10	− 0.67	2.3
TiAu10-10m	− 0.70	2.2
T-F180	− 0.79	163
TAu10-F30	− 0.67	2.8
TAu10-F60	− 0.68	3.1
TAu10-F120	− 0.68	3.2
TAu10-F180	− 0.72	13.6
TAu10-F240	− 0.77	173.0

Regarding the difference in the materials that underwent the pulsed and continuous thermal treatment—it results from the nature of both annealing approaches. In the case of furnace annealing, the whole sample is heated uniformly and any surface melting of titania NTs was not detected. On the other hand, when the laser treatment was applied, the thermal energy is mostly accumulated in the top part of the titania, causing not only gold nanoparticle formation, but also a change in the morphology of the nanotubes’ surface area. Since the electrochemical process occurs at the electrode/electrolyte interface, we believe this change in the surface zone plays a very important role. As was listed in Table 3, the thermally treated titania (TiAu10-10m) exhibits a much lower number of donor density compared to the TAu10-F180 and TAu10-F240. Therefore, we believe that the laser-induced changes in the material structure (defects such as oxygen vacancies^58^) especially at its topmost region, highly promote overall enhanced electrochemical response.

## Conclusion

In this work, a facile and rapid method of Au-coated titanium dioxide nanotubes modification which leads to enhancement of the photoactivity of the studied material is presented. TiO_2_NTs were formed in an optimised anodisation process which was further followed by calcination in a furnace to ensure an anatase crystalline phase. After the deposition of an Au layer, the material underwent treatment in pulse mode by means of UV laser. SEM inspection revealed the formation of Au nanoparticles on the rims of the tubes for mild irradiation conditions (i.e. 30 mJ/cm^2^ fluence). Increasing of laser beam energy led to melting of the top layer of the nanotubes; nevertheless, underneath, the initial architecture of the TiO_2_NTs remained intact. Structural measurements confirmed the anatase phase of the material and no shift of the main anatase mode peak was observed. However, it should be mentioned here that the highest energy fluence may lead to degradation of the crystal structure. This observation is supported by the XRD data. The optical studies indicated that laser processing is causing the narrowing of the energy bandgap, which is of key importance for future applications in solar-driven processes. The performed electrochemical analysis indicated the most active photomaterial for both thicknesses of the gold layer. For the optimised, laser-modified 5 nm Au film deposited onto titania nanotubes, the current density registered in dark conditions exceeds 2 mA/cm^2^, while for the 10 nm Au coating—over 4 mA/cm^2^. When the materials were illuminated by simulated solar light, the current densities reach over 3.5 and almost 6 mA/cm^2^, respectively. It should be underlined that for the reference materials, those values were far below 0.5 mA/cm^2^ both in the dark and under UV–vis. It was also proven that the concentration of donor density increases with the laser fluence, and for TiO_2_NTs covered with a 10 nm Au layer and irradiated with 240 mJ/cm^2^ laser fluence, it drastically increases in comparison to the other coated samples. This supports the observations made on the basis of LV measurements, and indicates the superior synergistic effect arising when titania covered by gold film is treated by laser beam under optimised conditions. The authors believe that the Au–TiO_2_ nanomaterial with boosted photoactivity can find application in water splitting simulated by the Sun, as water decomposition was observed in the studied case.

## Supplementary information

Supplementary Information.
