# Supplementary material for: Anodic titania nanotubes decorated with gold nanoparticles produced by laser-induced dewetting of thin metallic films
Source: Sci Rep. 2020 Nov 25;10:20506. doi: 10.1038/s41598-020-77710-x (PMC7688952; doi:10.1038/s41598-020-77710-x)
Supplement: Supplementary file 1 — Supplementary Information. [file 41598_2020_77710_MOESM1_ESM.pdf]

# Anodic titania nanotubes decorated with gold nanoparticles produced by laser-induced dewetting of thin metallic films

Katarzyna Grochowska[1*], Nikolay Nedyalkov[2], Jakub Karczewski[3], Łukasz Haryński[1], Gerard Śliwiński[1] and Katarzyna Siuzdak[1]

[1]Centre of Plasma and Laser Engineering, The Szewalski Institute of Fluid-Flow Machinery, Polish Academy of Sciences, 14 Fiszera St., 80-231 Gdańsk, Poland

[2]Institute of Electronics, Bulgarian Academy of Sciences, 72 Tsarigradsko Shose Blvd., Sofia 1784, Bulgaria

[3]Faculty of Applied Physics and Mathematics, Gdańsk University of Technology, 11/12 Narutowicza St., 80-233 Gdańsk, Poland

*e-mail: kgrochowska@imp.gda.pl

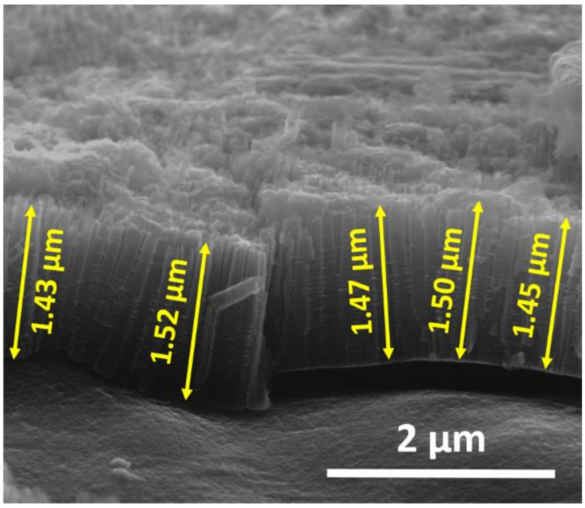


**Fig. S1.** SEM image (cross-section) of pristine $TiO_2$ nanotubes.

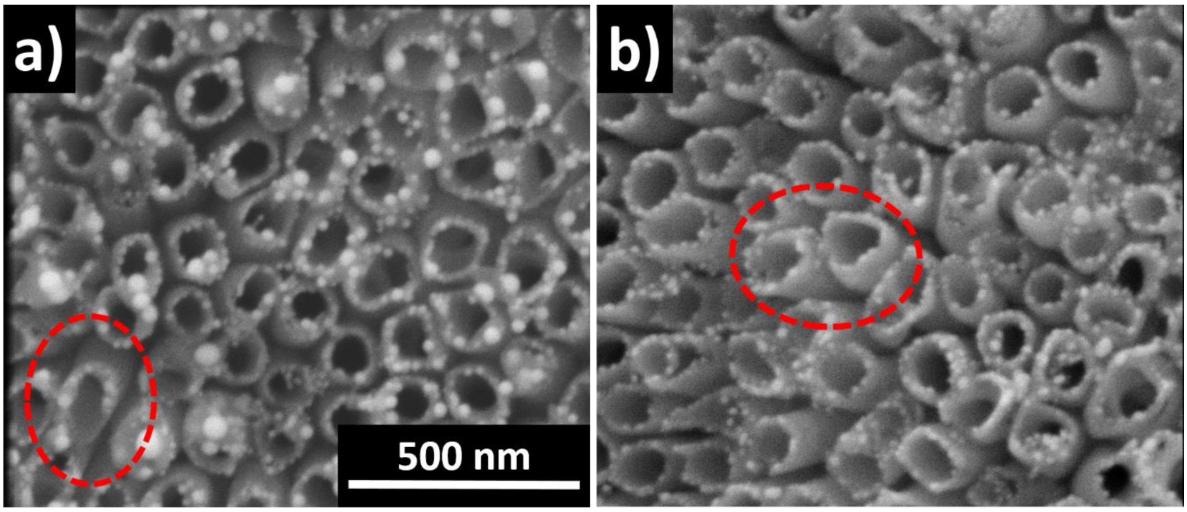


**Fig. S2.** SEM images of $TiO_2$NTs covered with 5 nm of Au layer treated with 30 mJ/cm$^2$ laser fluence (a) and in furnace for 10 minutes (b). Crown position of formed nanoparticles is additionally marked with red ovals.

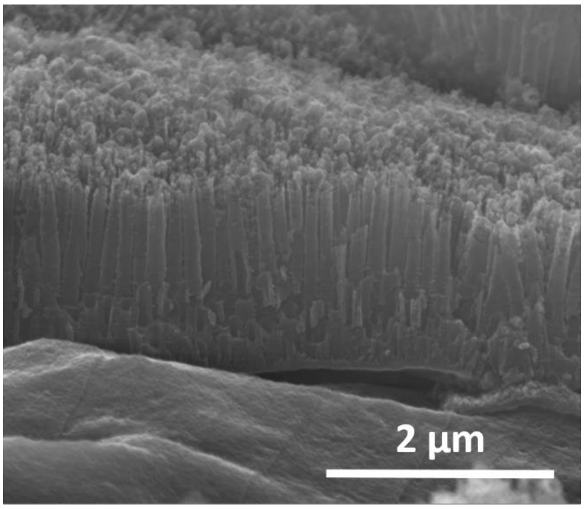


**Fig. S3.** SEM image (cross-section) of $TiO_2$ nanotubes covered with 10 nm of Au layer and laser treated with 30 mJ/cm$^2$.

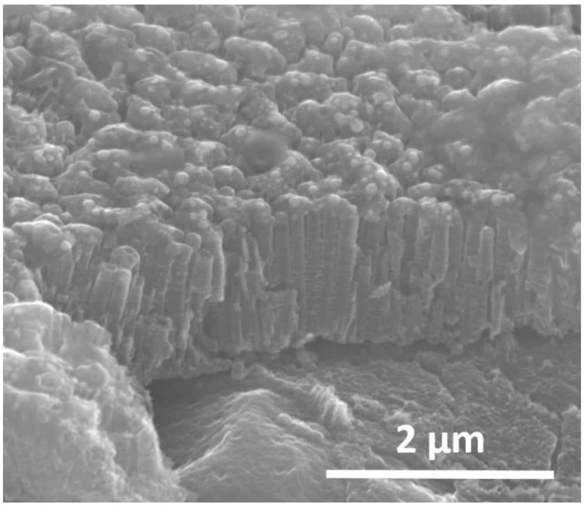


**Fig. S4.** SEM image (cross-section) of $TiO_2$ nanotubes covered with 10 nm of Au layer and laser treated with 180 mJ/$cm^2$.

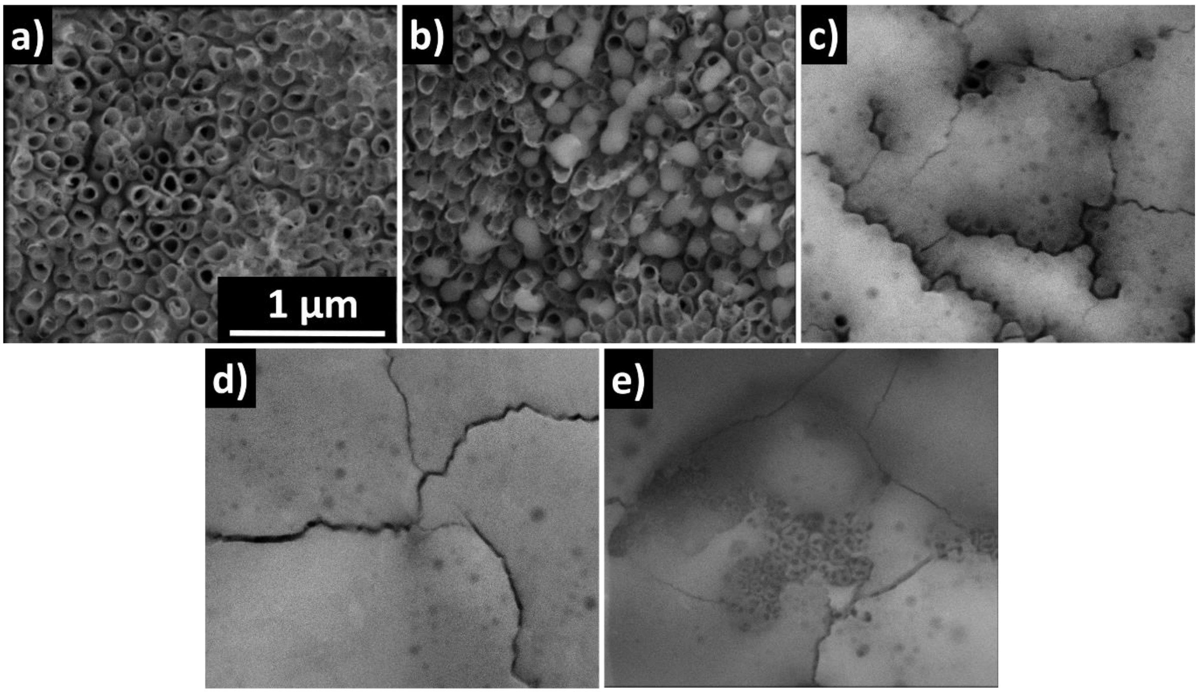


**Fig. S5.** SEM images of laser treated $TiO_2$NTs with fluences of 30 (a), 60 (b), 120 (c), 180 (d) and 240 (e) mJ/$cm^2$.

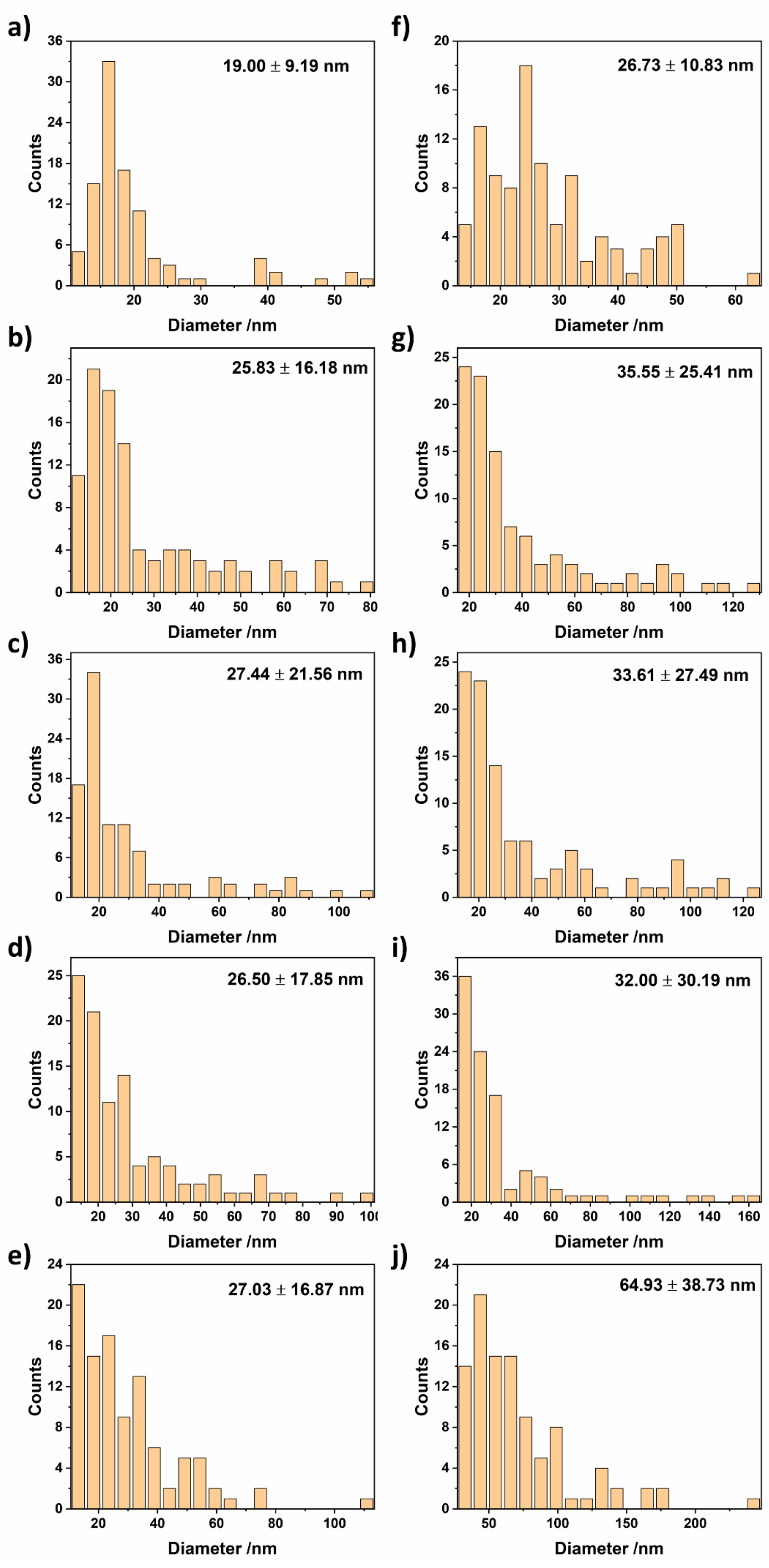


**Fig. S6.** Size distribution of Au nanoparticles obtained onto the $TiO_2$NTs initially covered with 5 (a-e) and 10 (f-g) nm Au layers and laser treated with fluences of 30 (a, f), 60 (b, g), 120 (c, h), 180 (d, i) and 240 (e, j) $mJ/cm^2$.

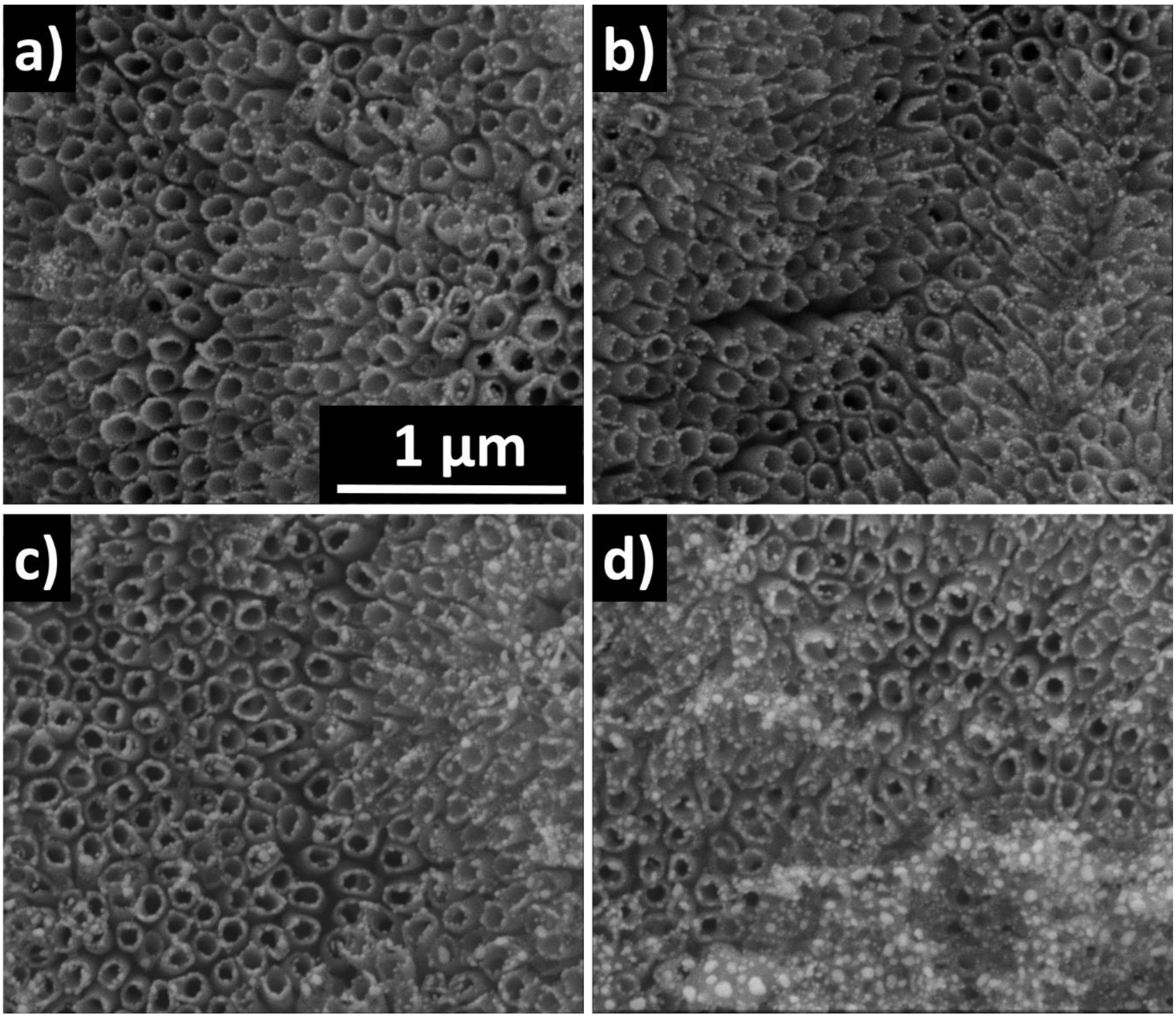


**Fig. S7.** SEM images of furnace treated $TiO_2$NTs covered with 5 (a, b) and 10 (c, d) nm Au films for 10 (a, c) and 30 (b, d) minutes.

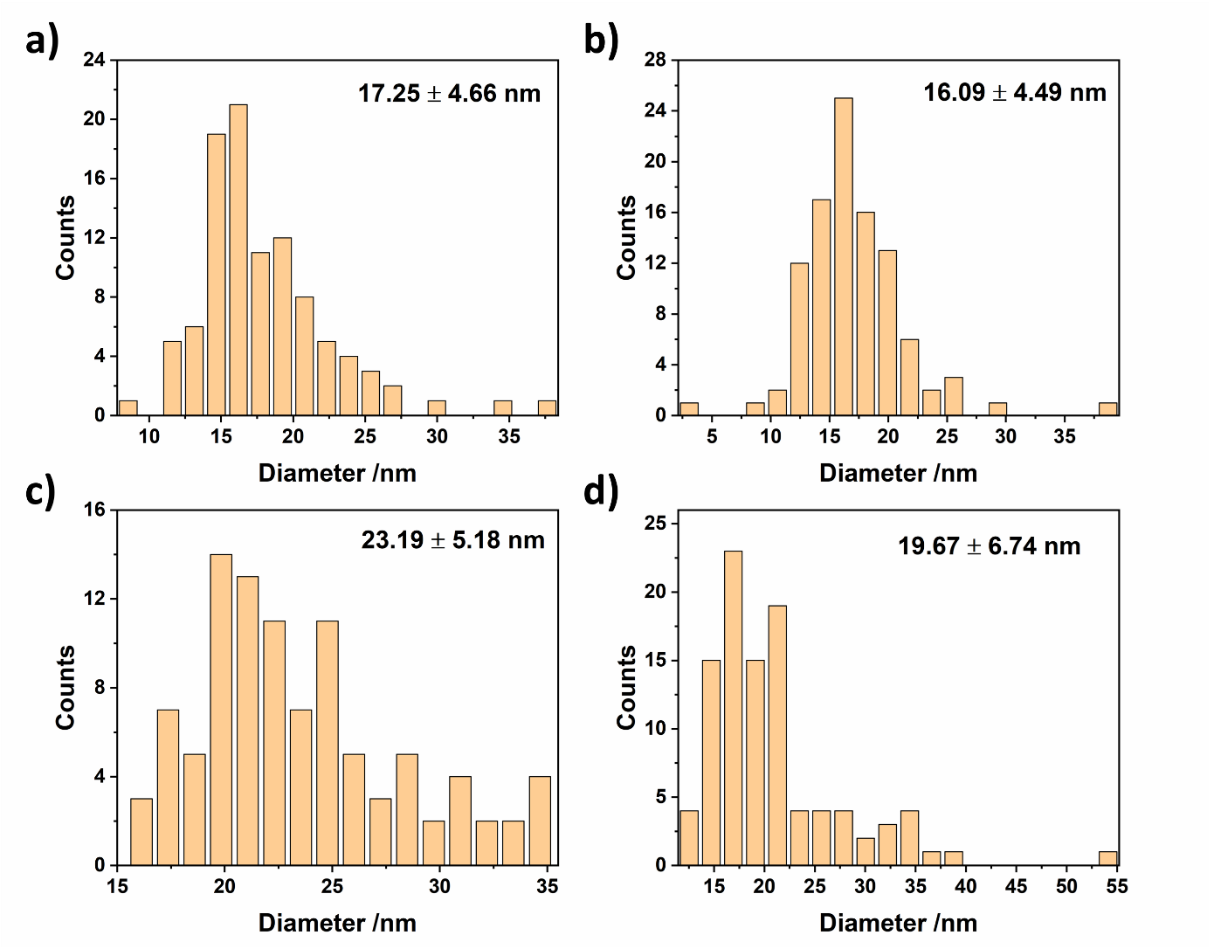


**Fig. S8.** Size distribution of Au nanoparticles obtained onto the $TiO_2$NTs initially covered with 5 (a, b) and 10 (c, d) nm Au films and treated in furnace for 10 (a, c) and 30 (b, d) minutes.

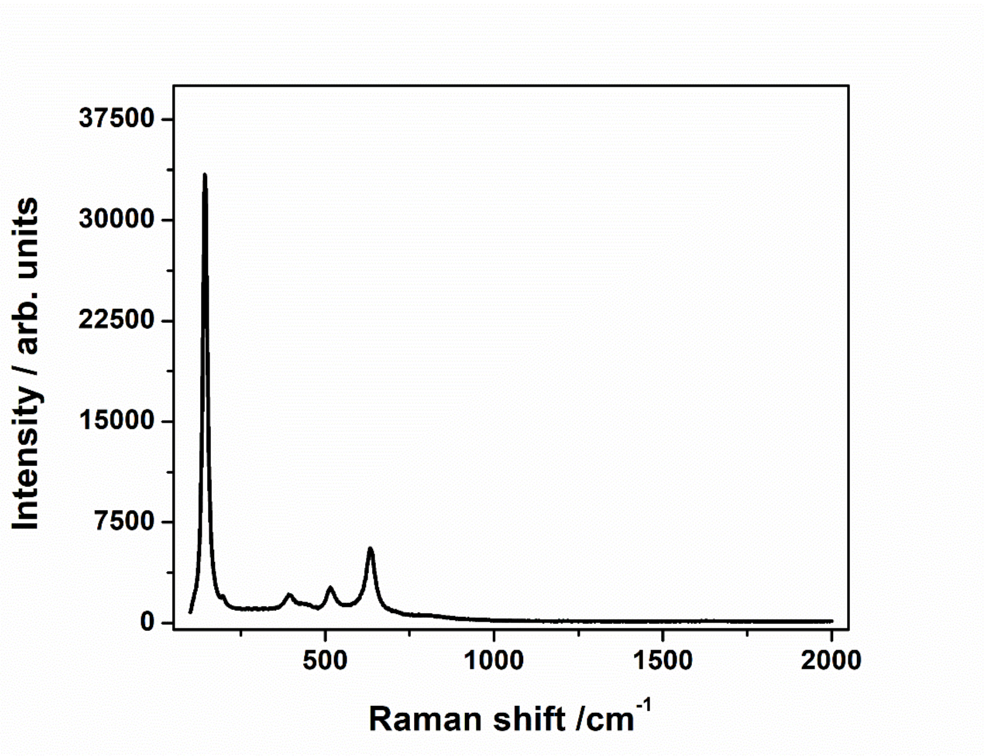


**Fig. S9.** Raman spectrum of bare $TiO_2$NTs.

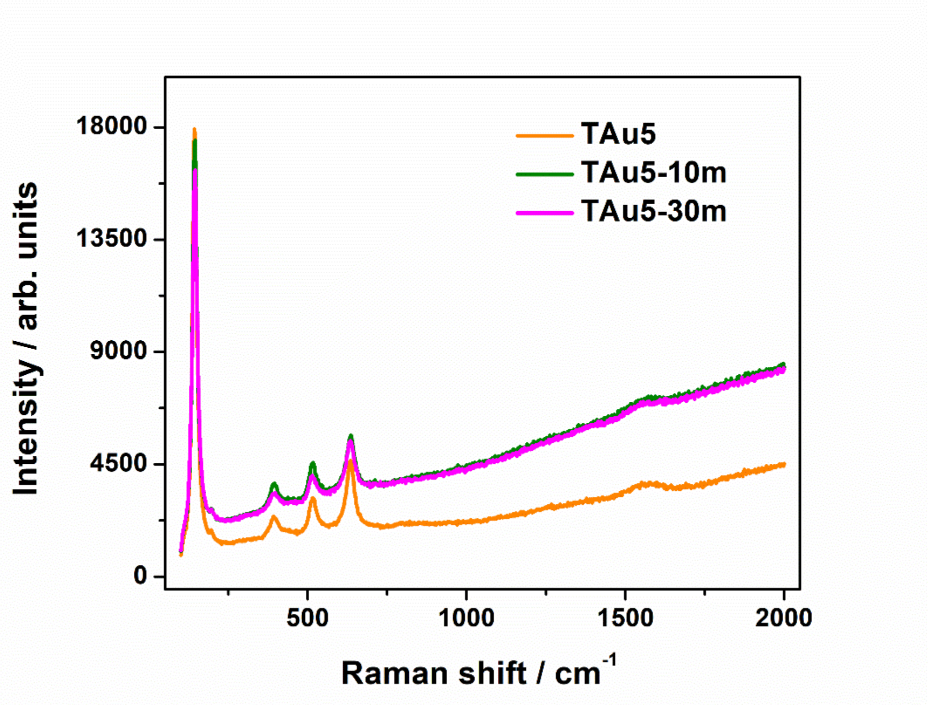


**Fig. S10.** Raman spectra of $TiO_2$NTs covered with 5 nm Au films before and after furnace treatment for 10 and 30 minutes.

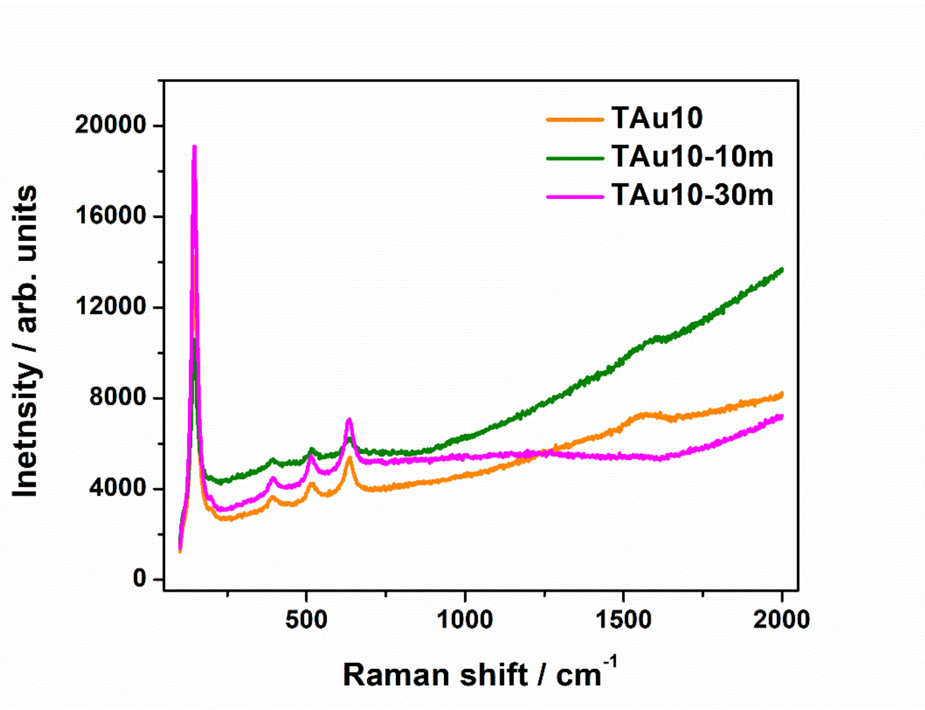


**Fig. S11.** Raman spectra of $TiO_2$NTs covered with 10 nm Au films before and after furnace treatment for 10 and 30 minutes.

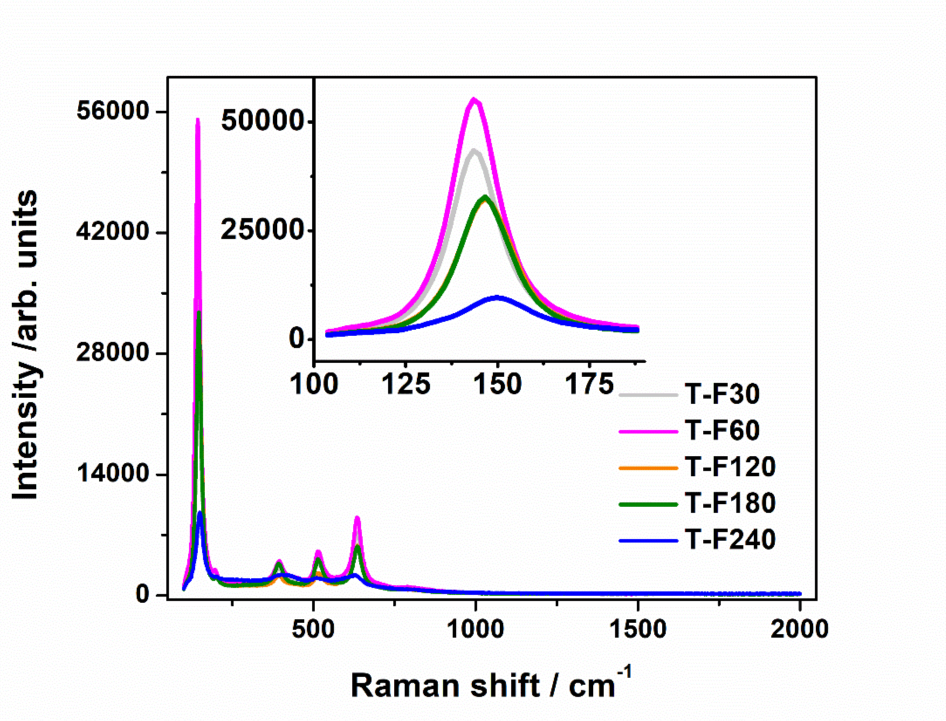


**Fig. S12.** Raman spectra of laser treated bare $TiO_2$NTs with fluences of 30, 60, 120, 180 and 240 $mJ/cm^2$.

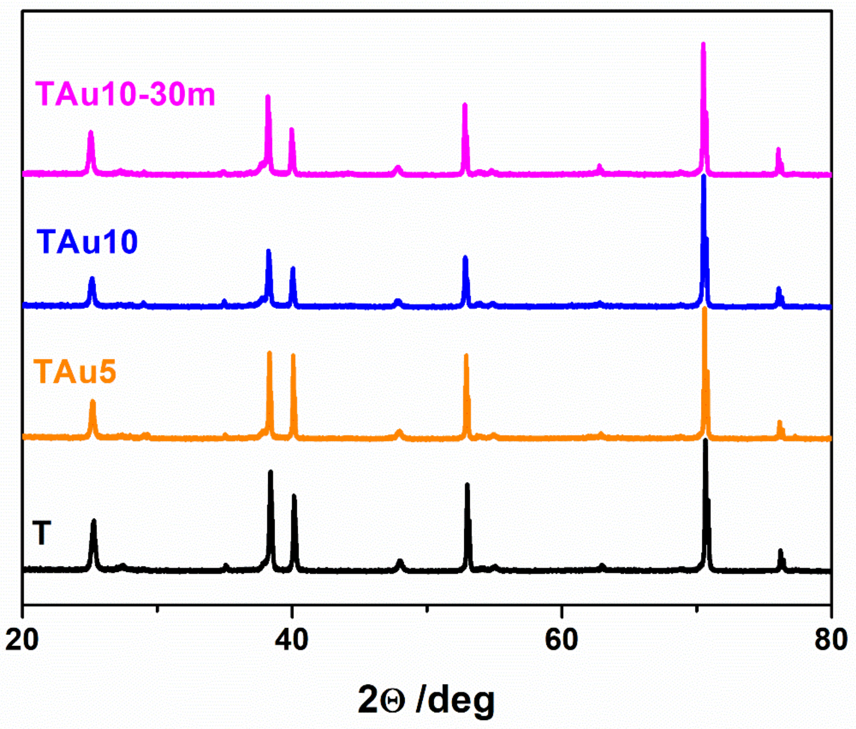


**Fig. S13.** XRD data of pristine titania, titania covered with gold layers (5 and 10 nm) and thermally treated in the furnace for 30 min.

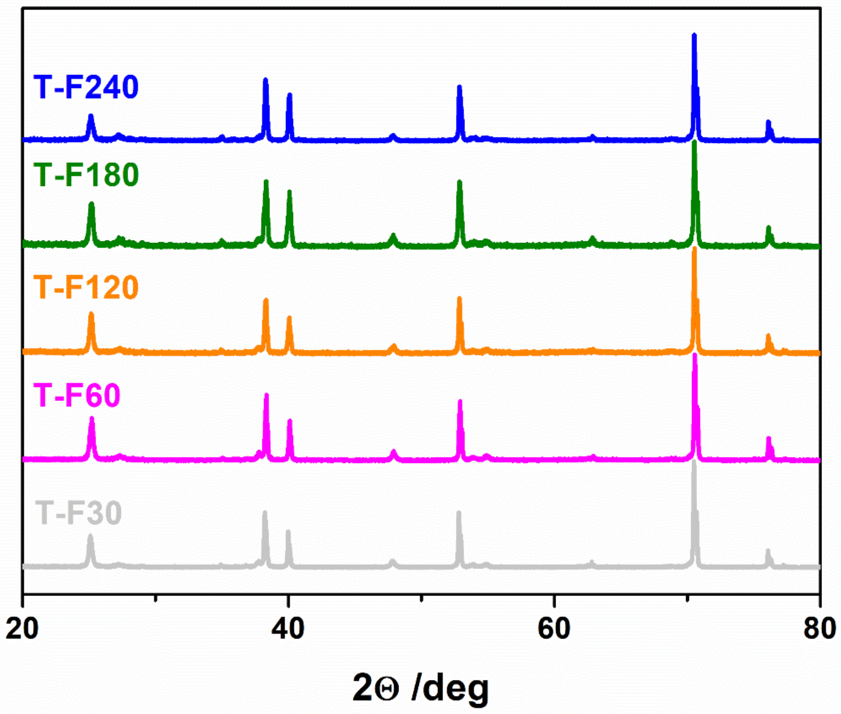


**Fig. S14.** XRD data of pristine titania treated with pulsed UV-laser of different fluence.

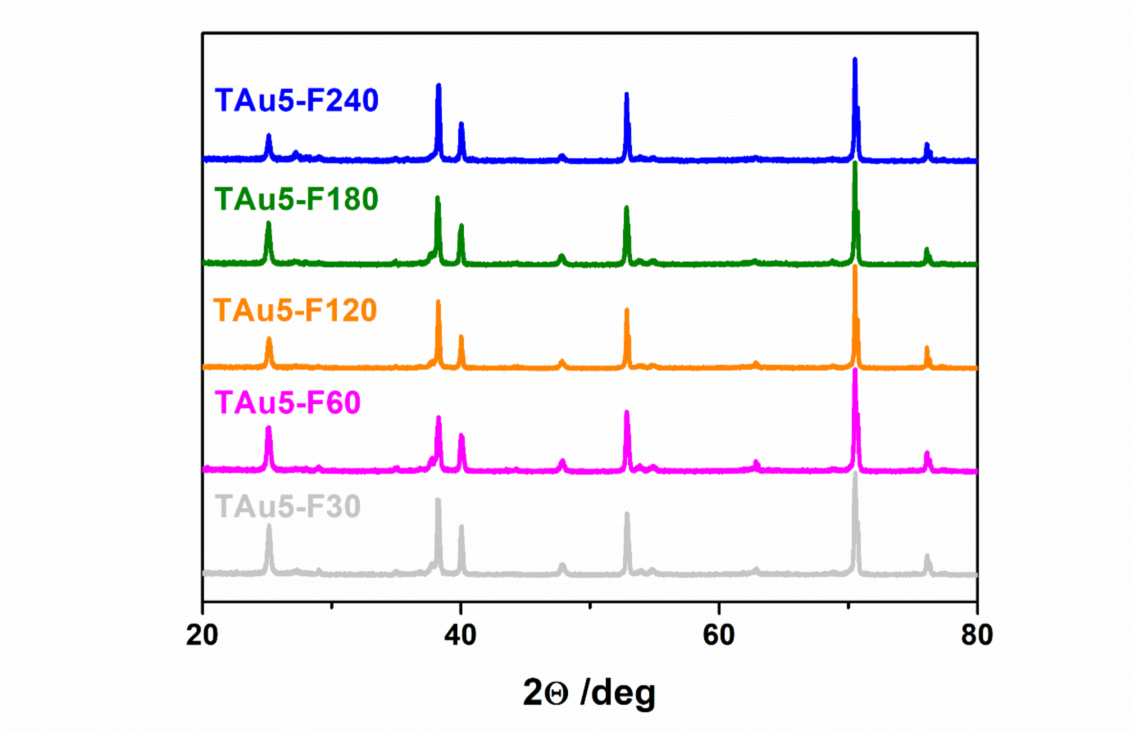


**Fig. S15.** XRD data of titania covered with 5 nm Au film and treated with pulsed UV-laser of different fluence.

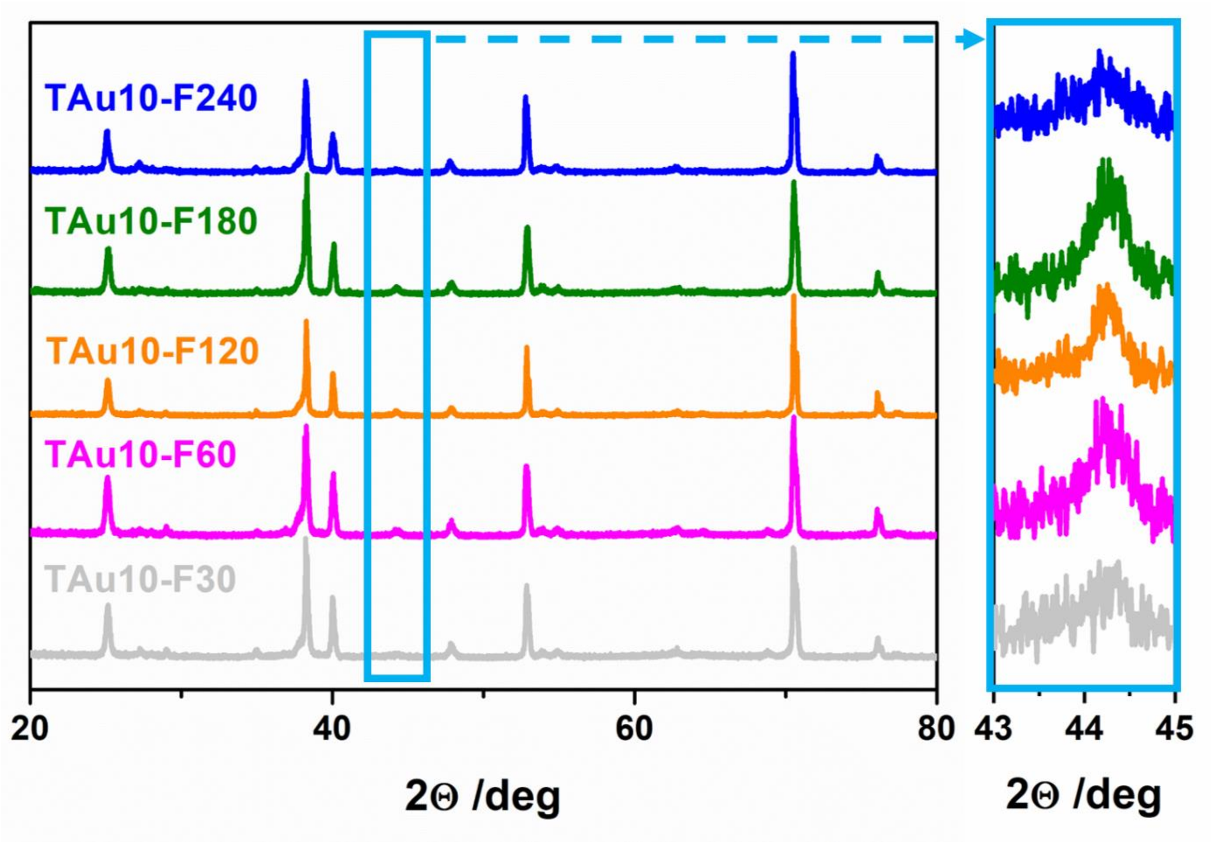


**Fig. S16.** XRD data of titania covered with 10 nm Au film and treated with pulsed UV-laser of different fluence.

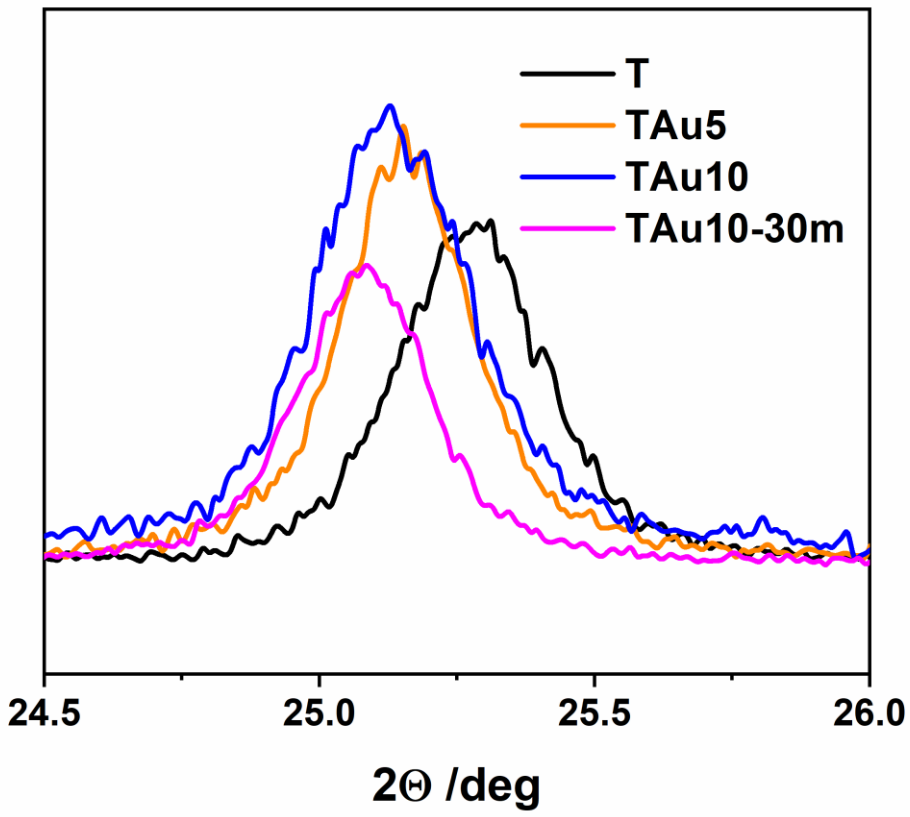


**Fig. S17.** The magnified view of anatase (101) peaks for pristine titania, titania covered with gold layers (5 and 10 nm) and thermally treated in the furnace for 30 min.

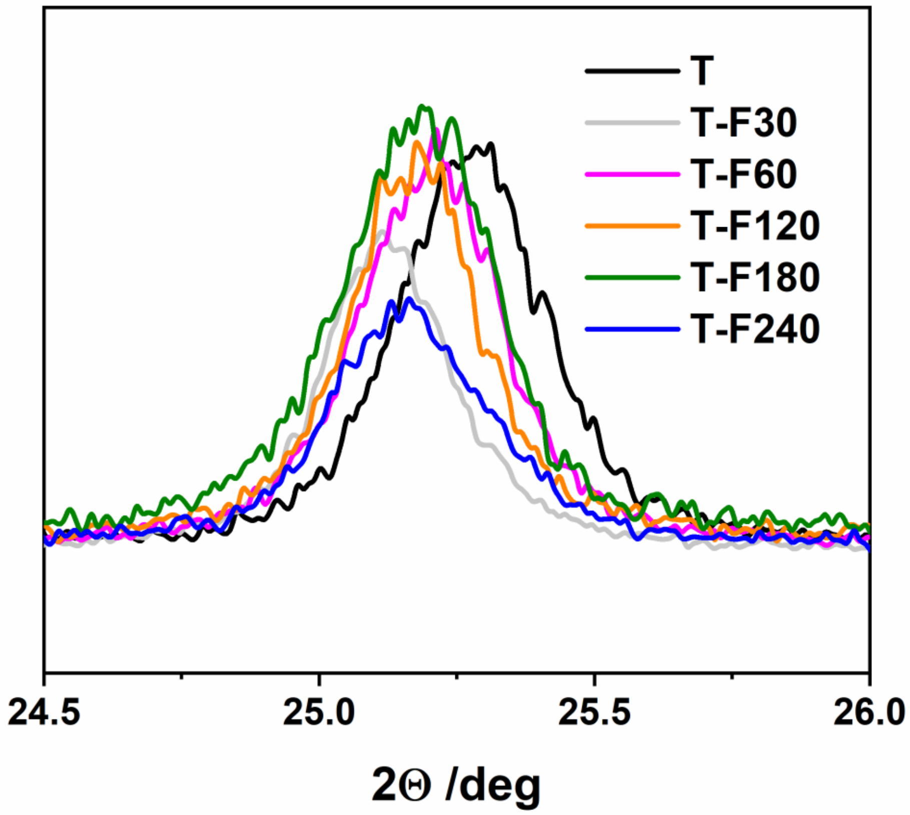


**Fig. S18.** The magnified view of anatase (101) peaks for pristine titania and pristine titania treated with pulsed UV-laser of different fluence.

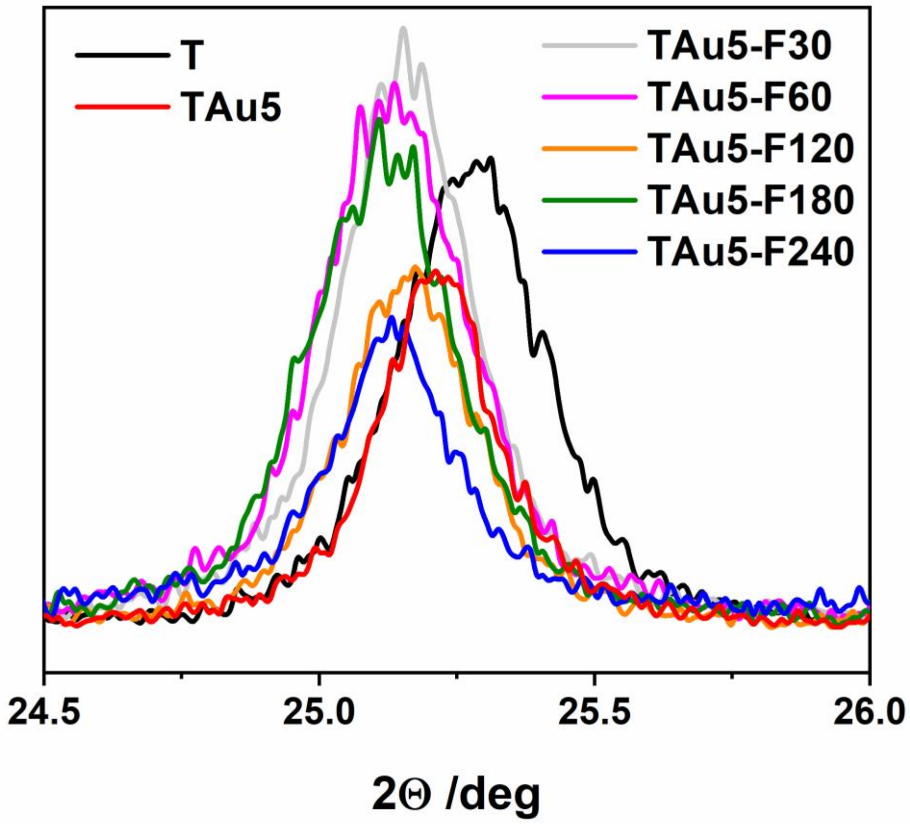


**Fig. S19.** The magnified view of anatase (101) peaks for pristine titania, titania covered with 5 nm gold layer and treated with pulsed UV-laser of different fluence.

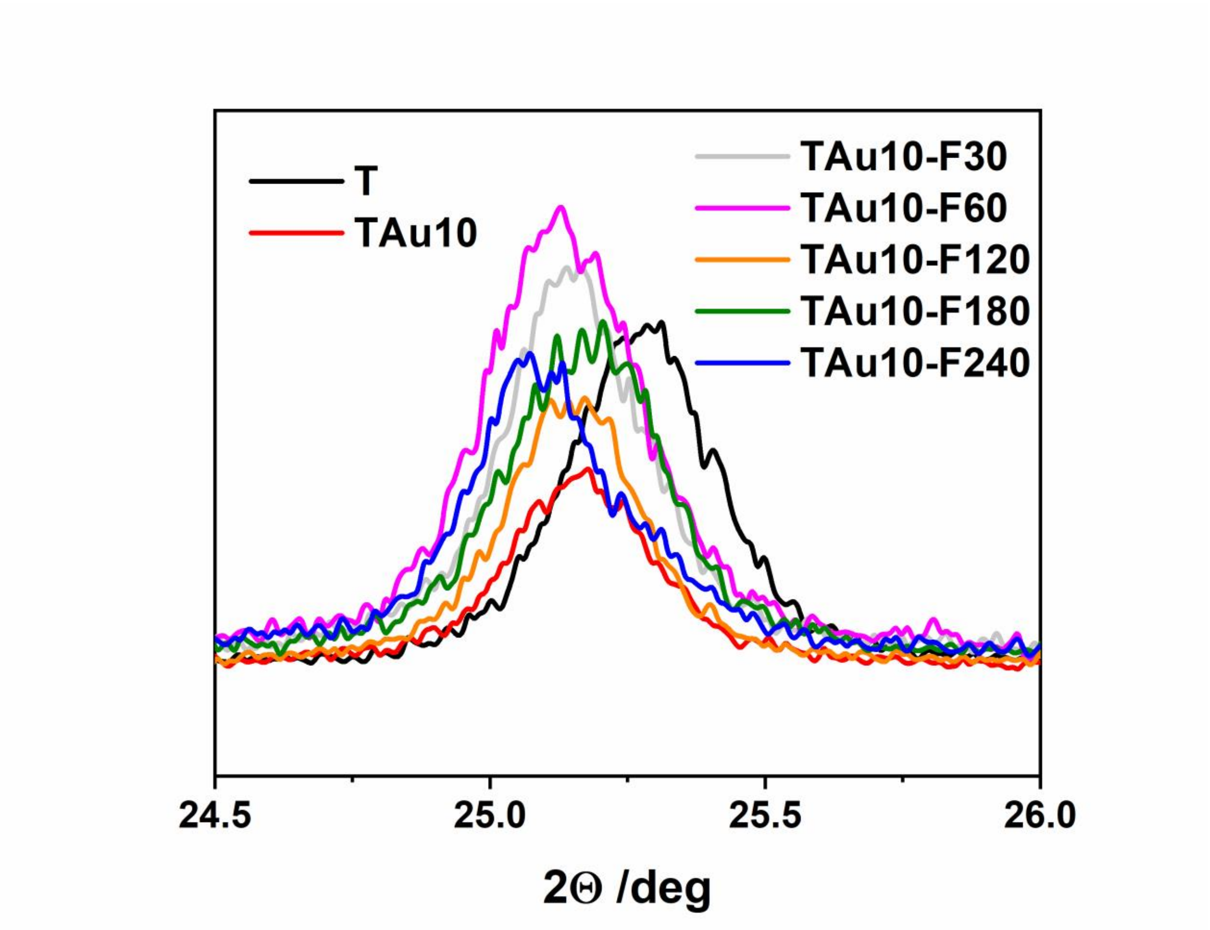


**Fig. S20.** The magnified view of anatase (101) peaks for pristine titania, titania covered with 10 nm gold layer and treated with pulsed UV-laser of different fluence.

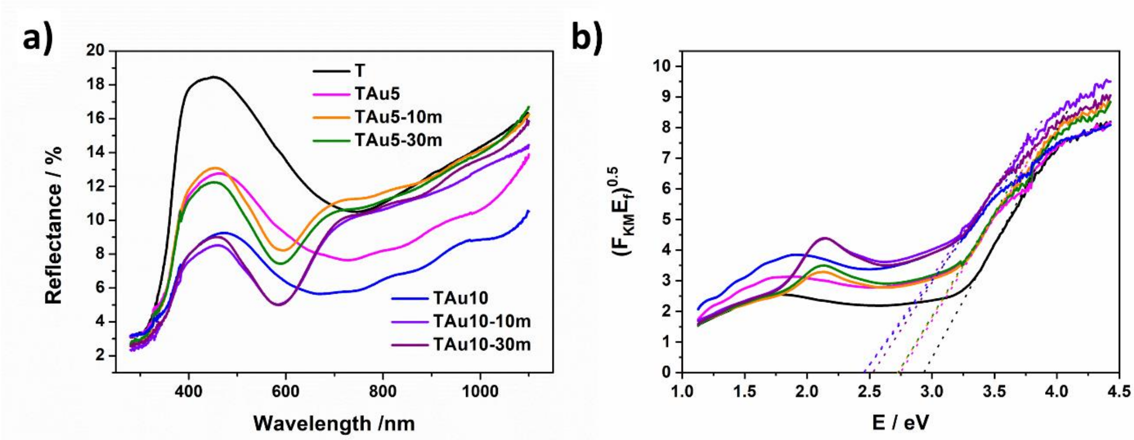


**Fig. S21.** Reflectance spectra (a) of bare $TiO_2$NTs and $TiO_2$NTs covered with 5 and 10 nm Au films before and after furnace treatment for 10 and 30 minutes and corresponding Tauc plots (b).

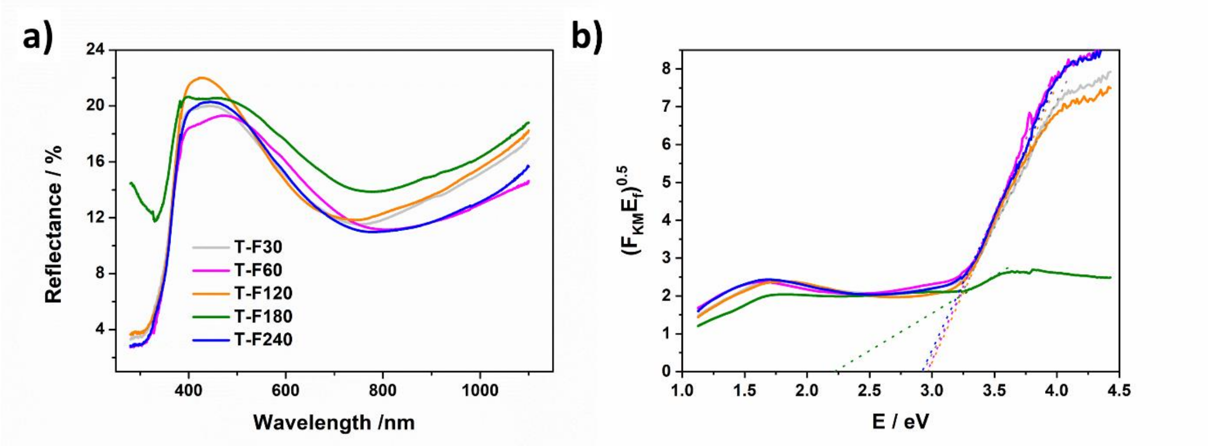


**Fig. S22.** Reflectance spectra (a) of laser treated bare $TiO_2$NTs with fluences of 30, 60, 120, 180 and 240 mJ/cm$^2$ and corresponding Tauc plots (b).

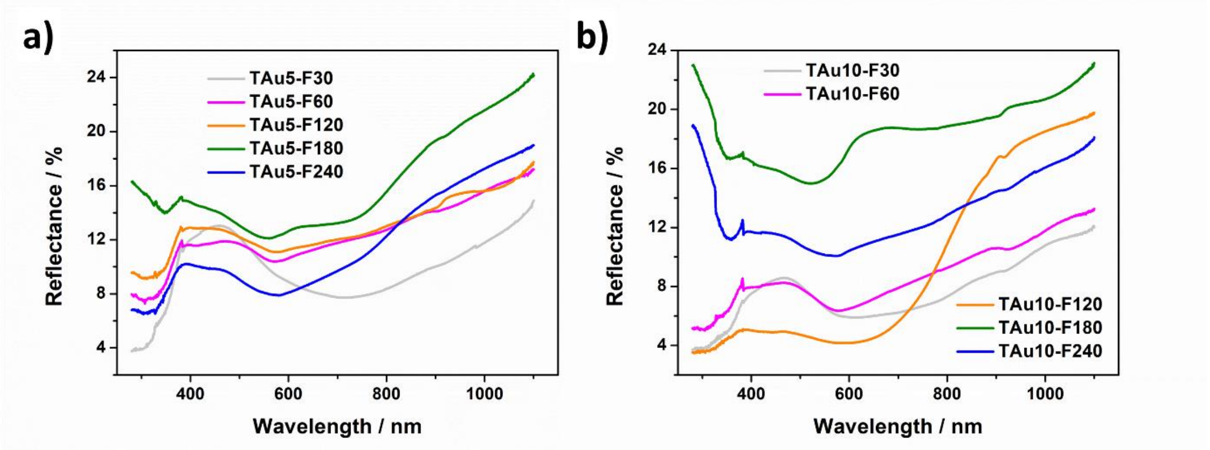


**Fig. S23.** Reflectance spectra of laser treated $TiO_2$NTs covered with 5 (a) and 10 (b) nm Au layers. Fluence kept in the range of 30-240 mJ/cm$^2$.

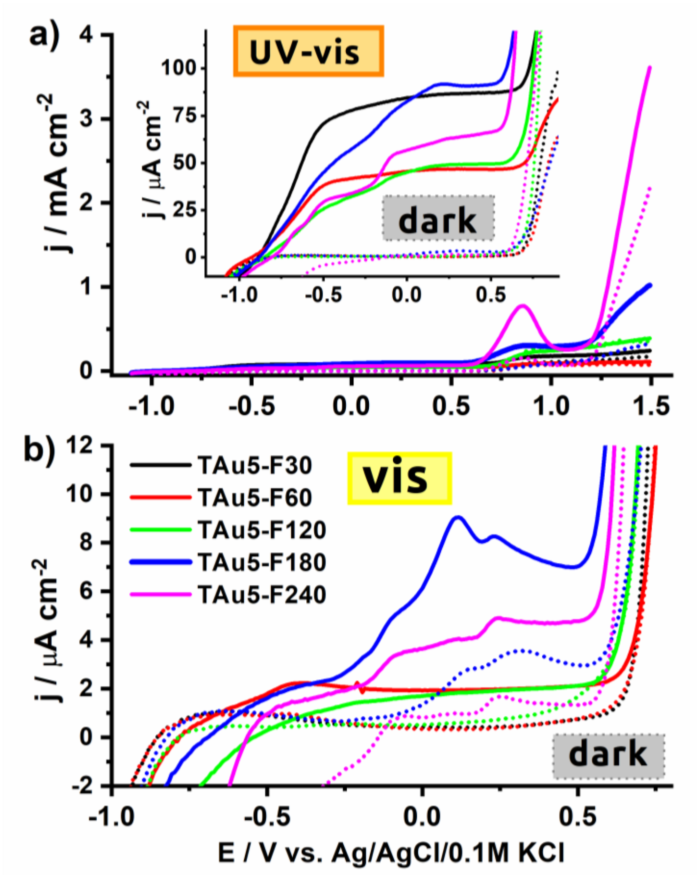


**Fig. S24.** Linear voltammetry curves recorded under a) UV-vis and b) vis irradiation for laser modified the samples with initially deposited 5 nm Au film.

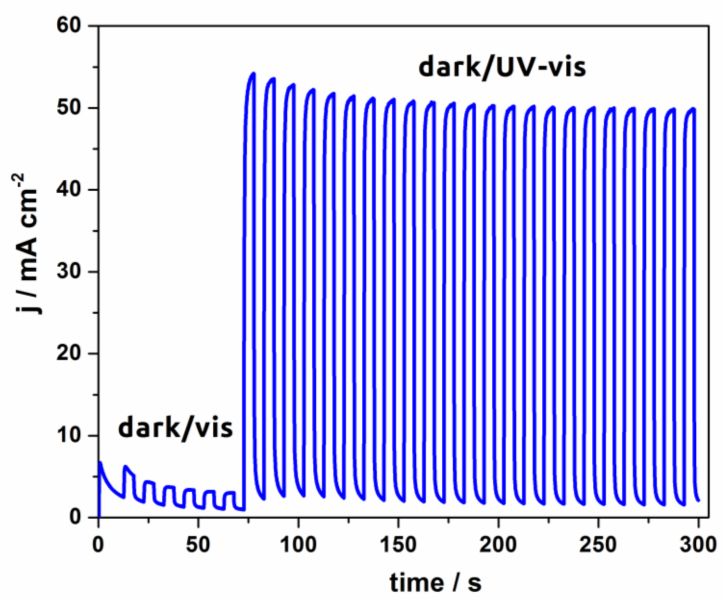


**Fig. S25.** The transient photocurrent recorded for TiAu-10-F240 sample under chopped vis and UV-vis irradiation.

**Table S1.** Current density values recorded at +1.5 V vs. Ag/AgCl/0.1M KCl under UV-vis irradiation.

| **Electrode material** | **j /mA cm$^{-2}$** | **Electrode material** | **j /mA cm$^{-2}$** |
|---|---|---|---|
| T | 0.14 | TAu-5-F30 | 0.24 |
| TAu5 | 0.15 | TAu-5-F60 | 0.11 |
| TAu10 | 0.47 | TAu-5-F120 | 0.38 |
| T-F30 | 0.14 | **TAu-5-F180** | **1.02** |
| T-F60 | 0.19 | **TAu-5-F240** | **3.62** |
| T-F120 | 0.28 | | |
| T-F180 | 0.25 | TAu-10-F30 | 0.13 |
| T-F240 | 0.31 | TAu-10-F60 | 0.29 |
| T-Au5-10m | 0.42 | TAu-10-F120 | 0.18 |
| T-Au5-30m | 0.10 | **TAu-10-F180** | **1.72** |
| T-Au10-10m | 0.46 | **TAu-10-F240** | **5.89** |
| T-Au10-30m | 0.26 | | |
